# Impact of Alkyl Spacer and Side Chain on Antimicrobial Activity of Monocationic and Dicationic Imidazolium Surface-Active Ionic Liquids: Experimental and Theoretical Insights

**DOI:** 10.3390/molecules29235743

**Published:** 2024-12-05

**Authors:** Marta Wojcieszak, Sylwia Zięba, Alina T. Dubis, Maciej Karolak, Łukasz Pałkowski, Agnieszka Marcinkowska, Andrzej Skrzypczak, Alicja Putowska, Katarzyna Materna

**Affiliations:** 1 Faculty of Chemical Technology, Poznan University of Technology, Berdychowo 4, 60-965 Poznań, Poland; marta.wojcieszak@put.poznan.pl (M.W.); agnieszka.marcinkowska@put.poznan.pl (A.M.); andrzej.skrzypczak@put.poznan.pl (A.S.); alicja.putowska@student.put.poznan.pl (A.P.); 2 Department of Molecular Crystals, Institute of Molecular Physics Polish Academy of Sciences, Smoluchowskiego 17, 60-179 Poznań, Poland; sylwia.zieba@ifmpan.poznan.pl; 3 Faculty of Chemistry, University of Bialystok, Ciołkowskiego 1K, 15-245 Białystok, Poland; alina@uwb.edu.pl; 4 Department of Pharmaceutical Technology, Faculty of Pharmacy, Nicolaus Copernicus University, Jurasza 2, 85-089 Bydgoszcz, Poland; maciej.karolak@cm.umk.pl (M.K.); lukaszpalkowski@cm.umk.pl (Ł.P.)

**Keywords:** micellization, microbes, surface tension, cationic amphiphiles, contact angle

## Abstract

This study investigates a series of surface-active ionic liquids (SAILs), including both imidazolium monocationic and dicationic compounds. These compounds are promising candidates, as they combine unique surface properties with antimicrobial activity, aligning with modern trends in chemistry. The research encompasses synthesis, thermal analysis, and topographical assessment, focusing on the impact of the amphiphilic cationic moiety, alkyl chain length, and the spatial relationship between the imidazolium ring and the phenyl substituent on the compounds’ physicochemical behavior. An added value of this work lies in the integration of theoretical calculations related to their behavior in solution and at the air–water interface, revealing spontaneous adsorption (negative Gibbs free energy of adsorption values, ΔG^0^_ads_). The results indicate that dicationic imidazolium SAILs have a greater tendency to form micelles but are less effective at reducing surface tension compared to their monocationic counterparts. Topography analyses of SAILs with 12 carbon atoms further highlight these differences. Notably, the dicationic SAIL with 12 carbon atoms in the spacer exhibited an impressive MIC of 0.007 mmol L^−1^ against *Candida albicans*, consistent with findings showing that dicationic SAILs outperformed conventional antifungal agents, such as amphotericin B and fluconazole, at equivalent concentrations. Overall, the synthesized SAILs demonstrate superior surface activity compared to commercial surfactants and show potential as disinfectant agents.

## 1. Introduction

The behavior of imidazolium surface-active ionic liquids (SAILs) in solution is of immense interest in both fundamental studies and applied research areas, such as cosmetics, adhesives, detergents, pharmaceutical products, and many other applications [1,2,3,4]. Moreover, the wide range of applications for imidazolium SAILs stems from their unique ability to form structures that combine ion pairs, imparting amphiphilic (hydrophobic–hydrophilic) characteristics to these compounds [5].

Maintaining the hydrophilic–hydrophobic balance, and thus the compound’s propensity to arrange itself in solution in an oriented manner to form micelles, is possible by functionalizing the compound’s structure [6,7,8,9,10]. One of the most common solutions is the introduction of an additional cation with an amphiphilic structure [11,12]. As a result, besides monocationic SAILs, there are also dicationic and tricationic imidazolium SAILs [13,14,15]. To date, the most extensively studied cation is 1-alkyl-3-methylimidazolium [10,16]. However, a review of the literature reveals the existence of other cations, such as 1-alkyloxycarbonyloxyethyl-3-methylimidazolium [17].

In the case of imidazolium-based surface-active compounds, a frequently described property is their antimicrobial activity, in which the length of the alkyl chain plays a crucial role. Florio et al. [18] emphasized that within a series of such compounds, two distinct regions can be identified. The first pertains to compounds with an alkyl chain length of 12 or 14 carbon atoms, as these SAILs exhibit the highest antimicrobial activity. In contrast, SAILs with alkyl chains containing more than 16 or fewer than 10 carbon atoms show a significant reduction in their ability to combat microorganisms [19,20]. Furthermore, Florio et al. [18] suggested that, in addition to the effect of alkyl chain length, the chemical structure of the amphiphilic part is equally important, for example, the presence and number of polar groups in the hydrophilic moiety.

Continuing on, in addition to changing the number of amphiphilic centers, by structural functionalization is meant the elongation of the alkyl side chain or spacer, which are located in the cation [21,22]. In the case of the hydrophobic spacer in di- or more cationic SAILs, the length of the alkyl spacer is crucial, as it affects the behavior of the compound molecules [23]. A distinction can be made between rigid, semi-rigid, and flexible spacers [24]. A rigid spacer usually contains an unsaturated double/triple bond [25] or an aromatic ring [26,27,28]. A flexible spacer, on the other hand, contains a long alkyl chain consisting of only methylene groups or ethoxy groups [24,29,30]. A semi-rigid spacer can be formed by a combination of flexible and inflexible groups [31]. Another way to influence the aggregation of SAILs is by inserting a functional ester, ether, or amide group into the side chain, which leads to significant changes in their ability to micellize. Moreover, one solution employed by scientists is to compare the hydrophobic and steric effects of imidazolium SAILs with those of polar groups, such as pyrrolidinium or pyridinium, as studied by Wang et al. [32] and Kamboj’s group [33]. In contrast, an increasing number of studies have ocused on the effect of the counterion on micellization of imidazolium SAILs, especially with regard to the 1-alkyl-3-methylimidazolium cation [17]. Singh et al. [34], on the other hand, analyzed the aggregation behavior of 3-hexadecyl-1-methylimidazolium cations with various aromatic counterions. Meanwhile, Ma et al. [35] examined imidazolium SAILs with anions such as anisate and bromobenzoate.

The aim of this study was to evaluate a prospective group of imidazolium chloride-based SAILs. Data in the literature [36] suggest that this class of compounds is primarily applied in biological contexts due to their high solubility and strong interaction with water molecules. To expand their potential applications, we synthesized new ionic combinations containing one or two amphiphilic cations. Our research focuses on analyzing the antimicrobial and surface properties of monocationic and dicationic imidazolium SAILs while also integrating theoretical calculations to enhance experimental studies. We hypothesize that elongating the side chain in monocationic SAILs and the alkyl spacers in dicationic SAILs are key factors that will lead to increased surface activity. Similarly, the microbiological activity of these compounds is expected to depend on the length of their hydrophobic components. However, determining the optimal number of carbon atoms in the hydrophobic region is essential, as this balance will yield compounds with both maximum microbiological and surface activity, making them suitable candidates for industrial disinfectants. Moreover, previous publications have highlighted that imidazolium SAILs possess superior surface activity compared to conventional surfactants, such as didecyldimethylammonium chloride (DDAC) [36,37]. Therefore, we aim to explore whether the synthesized compounds could effectively replace traditional surfactants in industrial applications. The second point, related to the mentioned publications, concerns the structure of SAILs and raises the issue of whether increasing the number of carbon atoms in the spacer between the imidazolium and phenyl substituent (at the N1 position in the imidazolium ring) will influence the surface and biological activity of the compounds.

## 2. Results and Discussion

### 2.1. Synthesis

Ten imidazolium compounds (monocationic and dicationic SAILs) were prepared through synthetic steps according to the following description. The molecular structure of these SAILs is shown in Figure 1. ^1^H NMR spectra were recorded on a Varian VNMR-S spectrometer (Agilent, Milan, Italy) operating at 300 MHz with tetramethylsilane as the internal standard. ^13^C NMR spectra were obtained with the same instrument at 75 MHz. CHN elemental analyses were performed at Adam Mickiewicz University, Poznan (Poland). All the chemical shifts (δ) of the studied monocationic and dicationic SAILs are shown in Figures S3–S24.

**Scheme 1:**
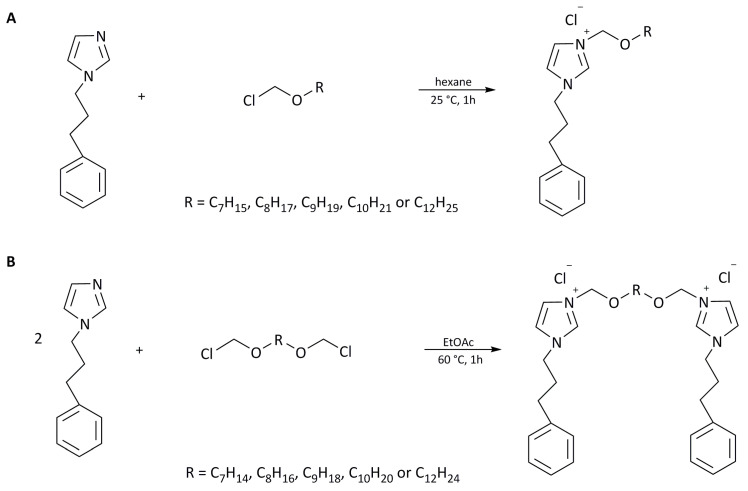
Synthesis of monocationic (**A**) and dicationic (**B**) imidazolium chlorides.

Monocationic imidazolium chlorides and dicationic imidazolium chlorides were obtained via quaternization reaction according to Scheme 1A,B.

**Preparation of 3-alkoxymethyl-1-(3-phenylpropyl) imidazolium chlorides (Scheme 1A)**

To a stirred solution of 1-(3-phenylpropyl) imidazole (0.1 mol) in anhydrous hexane (30 mL), the corresponding chloromethylalkyl ether (0.105 mol), freshly distilled under reduced pressure, was added dropwise at room temperature (25 °C). The solution was stirred at room temperature for 1 h. The obtained chlorides were washed with hexane. Subsequently, the hexane was removed under reduced pressure, and final products were dried at 60 °C under reduced pressure for 24 h. Obtained products were stored in anhydrous conditions in a vacuum desiccator over P_4_O_10_.

**Preparation of 3,3′-[α,ω-(dioxaalkane)]bis [1-(3-phenylpropyl)imidazolium] chlorides (Scheme 1B)**

Bis-imidazolium dichlorides were obtained via the Menschutkin reaction according to Scheme 1B. α,ω-(chlorometoxy)alkane (0.1 mol) was added to a round-bottomed flask that contained a vigorously stirred mixture of 0.12 mol of 1-(3-phenylpropyl) imidazole in 20 mL of anhydrous ethyl acetate. The reaction mixture was stirred at 60 °C under a reflux condenser for 1 h. Then, 1 mL of methanol was added to the post-reaction mixture, and, after cooling, the purified product fell to the bottom of the vessel in the form of a viscous, sometimes solidifying liquid. After the separation of the layers, the procedure was repeated. Then, from the lower layer, the solvents were evaporated in a vacuum evaporator, and, finally, the chlorides obtained were dried in a vacuum dryer and stored in anhydrous conditions in a vacuum desiccator over P_4_O_10_.

The purity of the synthesized chlorides was determined using a direct two-phase titration technique (EN ISO 2871-1,2:2010) to measure the surfactant content. The purity ranged from 92% to 99% for monocationic compounds and 90% to 98% for dicationic compounds, with the remainder up to 100% being water. An analogous synthetic procedure was also applied in our previous works [2,38]. Yields of monocationic compounds were 90–95%, while yields of dicationic compounds were 90–93%.

### 2.2. Thermal Analysis

**Figure 2:**
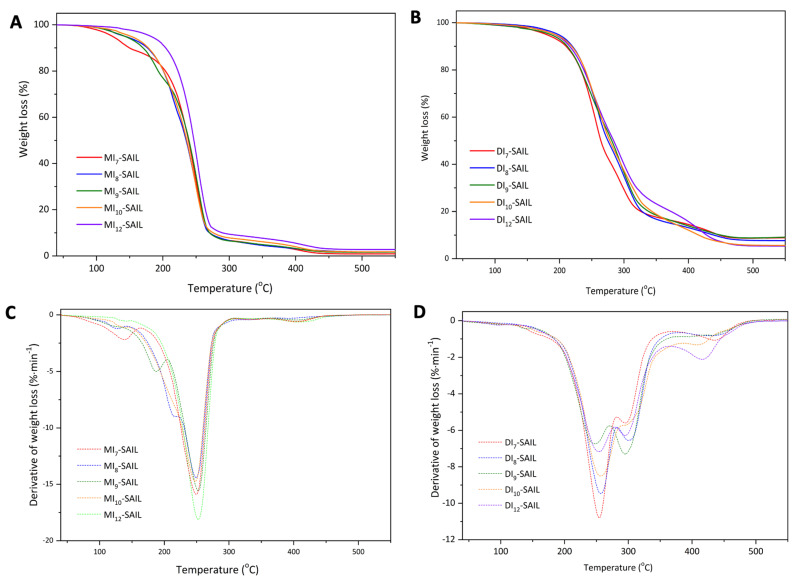
TG (**A**,**B**) and DTG (**C**,**D**) curves for a series of synthesized ionic liquids: (**A**,**C**) MI_n_-SAIL; (**B**,**D**) DI_n_-SAIL (where _n_ = 7, 8, 9, 10, and 12).

The thermal stability of the synthesized SAILs was evaluated using thermogravimetric analysis. The onset of thermal degradation was defined as the temperature at which 5% of the sample mass was lost (T_5%_), T_10%_ denoted the temperature of loss of 10% of the sample mass, T_50%_ denoted the temperature of loss of 50% of the sample mass, T_90%_ denoted the temperature of loss of 90% of the sample mass, and R denoted the residual mass. The thermal degradation data are summarized in Table 1, while the thermogravimetric (TG) and derivative thermogravimetric (DTG) curves for all the SAILs are shown in Figure 2. It was observed that the thermal decomposition of the monocationic SAIL series occurs at a lower temperature compared to the dicationic SAIL series. This observation is consistent with the reports presented by Hideaki Shirota et al. [14], who attribute the higher thermal resistance of dicationic ionic liquids to their higher density. We observed a similar relationship for a series of monocationic and dicationic ionic liquids of analogous structure differing in the length of the alkyl chain connecting the imidazolium ring to the phenyl substituent (at N1 position in the imidazolium ring) [2]. In previous studies, the spacer between imidazolium and the phenyl ring was ethylene; in this study, it was extended to propylene. This caused a slight decrease in the thermal resistance of the tested ionic liquids, e.g., in the MI-SAIL series the T_5%_ range dropped from 169.1–195.8 °C to 124–182 °C. Additionally, it seems that SAILs with an odd number of carbon atoms in the alkyl substituent, both in the case of monocationic and dicationic SAIL series, are characterized by slightly lower temperatures at which 5% of the sample decomposes. The monocationic SAIL series decomposes mainly in one step (the peak temperature of the maximum degradation rate estimated from the DTGs of all MI-SAIL curves is approximately DTG ≅ 250 °C); however, an additional step at lower temperatures can be observed, which with the increasing chain length of the alkyl substituent shifts to higher temperatures and disappears for **MI_12_-SAIL**. This may indicate that increasing the alkyl chain length of the substituent increases the resistance of the discussed SAILs. Moreover, as was mentioned above in the case of mono-SAIL, the effect of the alkyl chain length of the substituent on the initial thermal resistance of the tested compounds is visible, i.e., for 5% and 10% mass loss, the difference between the temperatures T_5%_ and T_10%_ of the **MI_7_-SAIL** and **MI_12_-SAIL** classes is about 50 °C, which is significant. However, further decomposition occurs at similar temperatures. For example, 50% decomposition is observed at nearly the same temperature for all SAILs: T_50%_ = 239 ± 5 °C. Therefore, the increase in the length of the substituent chain in the case of this series of ionic liquids increases the thermal resistance (the longer the chain, the higher the temperature needed to initiate its decomposition). However, once decomposition begins, it proceeds similarly for all liquids in the series. On the other hand, the series of dicationic SAILs decomposes mainly in two stages (DTG_1_ ≅ 250 °C and DTG_2_ ≅ 300 °C), with an additional decomposition stage observed at higher temperatures, particularly visible for **DI_10_-SAIL** and **DI_12_-SAIL**. Additionally, in the case of this series of SAILs, the influence of the alkyl spacer chain length is visible over the entire range of sample degradation (up to 90% mass loss), which is different to the case of monocationic SAILs. This may indicate decomposition of the alkyl chain in the first steps of degradation in the case of monocationic SAIL, while decomposition of the alkyl spacer in dicationic SAIL occurs in the later step of degradation.

The phase transition temperatures in the tested ionic liquids were determined from DSC thermograms. As mentioned in the research methodology, the glass transition (T_g_), recrystallization (T_cc_), and melting (T_m_) temperatures were determined from the heating cycle, while the crystallization (T_c_) temperatures were determined from the cooling cycle. In the first stage of the study, the sample was heated to 120 °C to remove moisture, which typically acts as a plasticizer for the glass transition temperature and, as a contaminant, also affects the melting and crystallization processes. The research results are shown in Table 1, while sample thermograms are shown in Figure S1 in SI. 

The glass transition temperatures for the monocationic SAIL series are characterized by higher values (from −19 °C to −35.2 °C) than the glass transition temperatures for the dicationic SAIL series (from −29 °C to −42 °C). In addition, the dependence of glass transition temperatures on the increase in the length of the alkyl chain for both SAIL series is different. This results from a significant difference in the structure of the tested SAIL series. In the case of monocationic liquids, the substituent is an increasingly longer alkyl chain, which can have a plasticizing effect on the tested ionic liquids by preventing vitrification. And with the increase in the length of the alkyl chain of the substituent from C8 to C10, we observed a decrease in the glass transition temperature, while for the C12 chain the glass transition temperature increased again. The glass transition temperature was not determined for the **MI_7_-SAIL** liquid because it probably occurs in the melting temperature range, which is superimposed on the glass transition. On the other hand, in the case of dicationic liquids, the alkyl chain is a spacer between two imidazolium rings. And in this case, we observed an increase in T_g_ with the increase in the length of the alkyl chain of the spacer, i.e., stiffening of the ionic liquids. The glass transition temperature is the only thermal transition for **DI_7_-SAIL** and **DI_8_-SAIL**. For these two ionic liquids, no other phase transitions were recorded in the tested temperature range. Moreover, for SAILs of the monocationic and dicationic series (except **DI_7_-SAIL** and **DI_8_-SAIL**), several solid–solid changes, i.e., polymorphism, were observed. This indicates the occurrence of different crystalline forms of the ionic liquids studied and the transition from one crystalline form to another, more stable form. The increase in the melting temperature with the increase in the length of the alkyl chain of the substituent (spacer) was observed, which was also previously reported by Pernak et al. [39] for ionic liquids with two ether substituents. However, a decrease in the melting temperature with the increase in the length of the alkyl chain of the substituent (spacer) was observed by Hideaki Shirota et al. [14], who studied monocationic and dicationic ionic liquids with alkyl substituents of different lengths (without an ether spacer and without an additional substituent in the N1 position, as in this study). Therefore, the results suggest that the presence of an ether atom may have a significant effect on the discussed transformation.

### 2.3. Surface Properties

Theoretically, the entire process illustrating micelle formation was detailed in our recent works [2,38,40,41]. Therefore, in this publication, we will focus on the key aspects of the behavior of the synthesized compounds in water, which ultimately contribute to the micellization process. Figure S2 in the SI presents the surface tension (γ) versus the logarithm of concentration (log C) for the synthesized SAILs at a constant temperature of 25 °C. The micellization process involves several key stages, starting with a noticeable decrease in surface tension for all SAILs, followed by a transition to a nearly constant surface tension. This behavior, as described by molecular self-organization theory, results from the formation of an amphiphilic monolayer at the air–SAIL interface. Once the interface is saturated with SAIL molecules, self-organized single molecules form, leading to the creation of micelles [42,43,44]. Furthermore, for each SAIL (monocationic and dicationic), the concentration at which micellization begins, referred to as the critical micelle concentration (CMC) [40], can be identified, as discussed later.

When analyzing the amphiphilic structure of the synthesized compounds, it is important to appreciate the role of the counterion [1,42,43], which belongs to the halides. In our case, chloride-based SAILs, although not larger or more polarizable than bromides, exhibit stronger micellar interaction in water [1].

The values of the parameters of the surface properties of the tested compounds are listed in Table 2.

The surface tension at CMC (γ_CMC_) of the monocationic and dicationic imidazolium SAILs decreased from the value of water (72.8 mN m^−1^) to a minimum ranging from 29.8 to 33.6 mN m^−1^ (monocationic SAILs) and from 38.6 to 46.8 mN m^−1^ (dicationic SAILs), at which point a plateau occurred. Notably, the differences between the lowest and the highest values for γ_CMC_ were almost 3.9 mN m^−1^ (monocationic SAILs) and 8.2 mN m^−1^ (dicationic SAILs). In the context of this situation, it can be concluded that, firstly, the γ_CMC_ values for compounds with one amphiphilic part are at the same level, and a similar conclusion can be drawn for compounds with two amphiphilic parts. Secondly, according to our recent work, the surface tension values during the micellization process are higher for dicationic SAILs than for monocationic SAILs [2]. On the other hand, when comparing the γ_CMC_ values of the synthesized monocationic SAILs, this parameter is significantly lower. An opposite trend was described by Ren et al. [48], who observed that molecules of bis-imidazolium compounds pack more closely than their monocationic analogs, which translates into lower γ_CMC_ values. The same suggestion was highlighted by Ao et al. [49], but in their studies the γ_CMC_ values of imidazolium ILs-type Gemini compounds were somewhat lower than those of monoimidazolium ILs. However, our data, presented in Table 2, clearly confirm that monocationic and dicationic SAILs exhibit surface activity, which is manifested by their tendency to form micelles. This phenomenon is clearly evident, as supported by the CMC values obtained. For monocationic SAILs, CMC values ranged from 38.54 to 1.32 mmol L^−1^ (from **MI_7_-SAIL** to **MI_12_-SAIL**), and for dicationic SAILs, they ranged from 47.35 to 0.28 mmol L^−1^ (from **DI_7_-SAIL** to **DI_12_-SAIL**). The CMC values obtained show compliance with the Klevens rule, according to which, generally, the CMC depends on the hydrocarbon atom chain length [50].

Looking at the obtained CMC results for monocationic and dicationic SAILs, a strong dependence can be observed between their tendency to micellize and the hydrophobicity of the compounds (see Table 2). Regarding the observed trend, the more carbon atoms in the alkyl spacer or alkyl chain, the lower the CMC value [51]. This issue will be discussed in detail in the subsequent sections of the paper. Baltazar et al. [12], as well as our own analysis [40], described the importance of the spatial arrangement of long alkyl groups, which has a crucial impact on micellization. In the case of this work, there is asymmetric substitution of alkyl substituents in monocationic SAILs, leading to an enhanced ability to form micelles at lower CMC values. For dicationic SAILs, on the other hand, the distance between the two amphiphilic parts is crucial. This distance, understood as the alkyl spacer, can influence the CMC values. The synthesized dicationic SAILs have a spacer made of carbon atoms and two oxygen atoms. This structure, on the one hand, allows flexibility (due to the alkyl chain), which affects the arrangement of the compound molecules into aggregates called micelles. On the other hand, the presence of oxygen atoms results in the stiffening of the compound molecules, as well as the potential for hydrogen bonding. Brycki et al. [52] emphasized that the introduction of an oxygen atom into the alkyl chain forming the spacer between the two amphiphilic moieties causes only minor changes, leading to lower CMC values. Moreover, the authors noted that the lower CMC values may be due to a reduction in Coulombic repulsion between the two cations, which influences the packing of the micelles [53,54].

**Figure 3:**
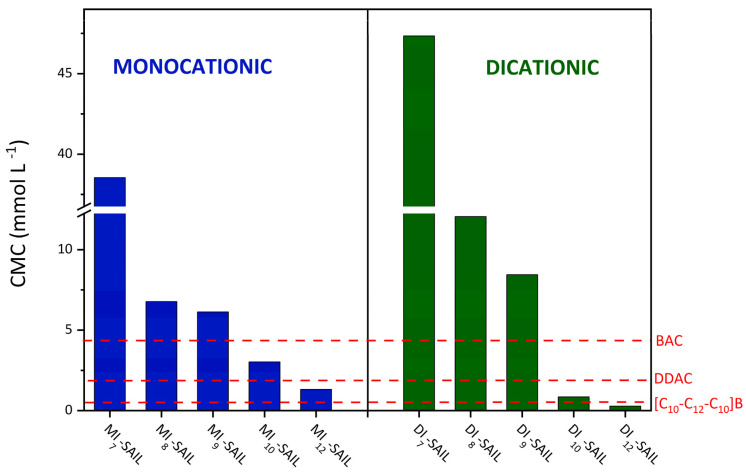
Comparison of the CMC values of the synthesized monocationic and dicationic SAILs with commercial surfactants, taking into account the chain length and the alkyl spacer.

Given the goal of the research to use the synthesized monocationic and dicationic SAILs as replacements for conventional surfactants, it was reasonable to compare the surface activity of these two groups of compounds. The surface activity of the tested SAILs was compared with that of commonly available cationic surfactants and widely used biocides, such as didecyldimethylammonium chloride (**DDAC**; Sigma-Aldrich, Burlington, Massachusetts, United States, purity ≥ 98%) and benzalkonium chloride (**BAC**; Sigma-Aldrich, Burlington, Massachusetts, United States, purity ≥ 95.0%). The compilation of the surface activity of these surfactants is particularly important because they were also used as references for microbiological studies in Section 2.5. In addition, Table 2 collects examples of cationic surfactants that are commonly used in industry (domiphen bromide (**DomphB**) and a homologous series of alkyltrimethylammonium bromides, namely, **C_10_TAB** and **C_12_TAB**). The above-mentioned cationic surfactants belong to monocationic compounds; therefore, their surface properties were compared with those of the structurally analogous synthesized SAILs (see Figure 3).

Based on the results which are presented in Figure 3, some of the synthetized SAILs (both monocationic and dicationic compounds) exhibited a greater tendency to self-aggregate and micellize than **DDAC** and **BAC**. Although **MI_12_-SAIL** has more carbon atoms in the alkyl chain, its CMC value is only slightly lower than that of **DDAC**. On the other hand, **C_10_TAB** and **C_12_TAB**, as representatives of conventional quaternary ammonium surfactants, have only one long alkyl chain in their structure. Therefore, it is easy to compare the CMC values of **C_10_TAB** with **MI_10_-SAIL** and **C_12_TAB** with **MI_12_-SAIL**. The CMC values of the pairs of compounds compared differ significantly and reflect the higher surface activity of the synthesized compounds. For the first pair, **C_10_TAB** and **MI_10_-SAIL**, the CMC value is almost 23 times higher for **C_10_TAB**, while, for the second pair, **C_12_TAB** with **MI_12_-SAIL**, the CMC value is over 11 times higher for **C_12_TAB**. In contrast, BAC contains only one long alkyl substituent, and the CMC value for this surfactant is higher than that of **MI_10_-SAIL** and **MI_12_-SAIL**. In the case of **DomphB**, its surface activity is only lower than **MI_12_-SAIL**, despite the fact that, like the synthesized SAIL, it contains one alkyl chain with 12 carbon atoms. Surfactants such as **BAC** and **DomphB**, as well as SAILs from **MI_8_-SAIL** to **MI_12_-SAIL**, have in common the aromatic ring as a substituent. Therefore, it should be emphasized that compounds containing aromatic rings as substituents in the amphiphilic part may exhibit higher surface activity compared to conventional surfactants, for which the delocalized charge of N^+^ weakens the electrostatic repulsion, thereby determining the self-aggregation of compounds [1]. The comparison of the surface activity of dicationic SAILs with commercial compounds containing double amphiphilic centers was conducted based on the data presented in Figure 3. The compound abbreviated as **[C10-C12-C10]B** and chemically named 1,10-bis(3-decylimidazolium-1-yl)dodecane dibromide, belonging to the cationic SAILs and containing 12 carbon atoms in the alkyl spacer, exhibits lower surface activity (indicated by a higher CMC value of 0.60 mmol dm^−3^) compared to its synthesized analog **DI_12_-SAIL** with the same spacer length.

**Figure 4:**
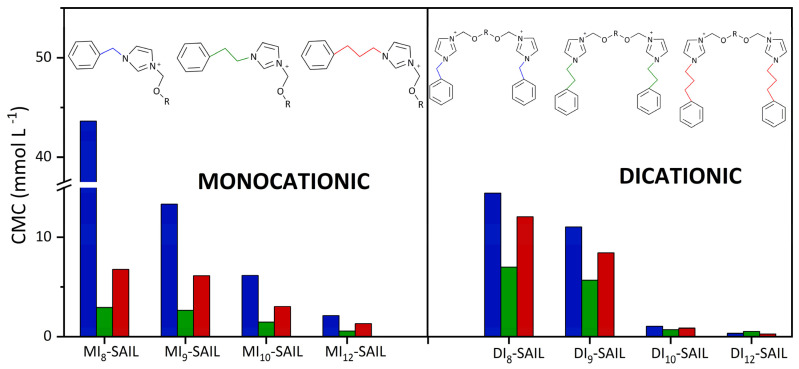
Summary of CMC values of synthesized compounds and selected series of imidazolium SAILs.

To highlight the comprehensiveness of the research on the synthesized compounds, a summary is provided (see Figure 4) that compares the CMC values of the series of imidazolium SAILs studied so far.

Based on the data presented in Figure 4, very interesting correlations can be observed, which help to determine the optimal structure of the compound that ensures its highest surface activity. So far, the effect of elongating the alkyl chain and the alkyl spacer on the surface properties of SAILs has been thoroughly discussed. All these considerations have been supported by data in the literature [1,3,6]. However, the question remains whether there is a relationship between the number of carbon atoms connecting the imidazolium ring with the phenyl substituent. In the analysis, one, two, and three carbon atoms located between the aforementioned imidazolium ring and the phenyl substituent were considered. The lowest surface activity, as evaluated by CMC values, was shown by the series of SAILs with one carbon atom. Interestingly, the highest surface activity was observed for the series of compounds with two carbon atoms. The surface activity of the series of compounds with three carbon atoms between the imidazolium ring and the phenyl substituent fell between the two previously mentioned SAILs. Therefore, it is concluded that elongating the so-called alkyl “spacer” does not decrease the CMC value as would be expected. Furthermore, it is hypothesized that increasing the number of carbon atoms in the spacer between the imidazolium ring and the phenyl substituent may not only promote micelle formation but may effectively prevent micellization from occurring. This proposal is of great interest and will be the focus of our future research.

In general, the adsorption efficiency values (pC_20_), as shown in Table 2, tend to increase with the number of carbon atoms in the hydrophobic chain, with the exception of **MI_8_-SAIL**. This trend is well-known in the literature and is often highlighted in studies without deeper interpretation [55]. However, based on our results, we aimed to address several specific aspects. The initial aspect was influenced by Rosen’s observation [43] that pC_20_ values frequently rise in a linear fashion as the alkyl chain lengthens, indicating the negative free energy associated with the adsorption of methylene groups at these interfaces. As reported by the aforementioned author, for cationic compounds with surface activity, the introduction of two additional carbon atoms into the alkyl chain leads not only to an increase in pC_20_ values for adsorption at the water–air interface but also indicates the saturation of the interface by these compounds. Theoretically, for cationic amphiphiles, the growth dependence of the increase in the alkyl chain of the part by two carbon atoms is accompanied by an increase in pC_20_ values by 0.56–0.60. Thus, when analyzing the **MI_8_-SAIL**, **MI_10_-SAIL**, and **MI_12_-SAIL** and the **DI_8_-SAIL**, **DI_10_-SAIL**, and **DI_12_-SAIL** series, the difference in pC_20_ values between the compounds is in the range of 0.12 to 1.17, which consistently shows that from the pC_20_ value we are unable to determine the % of the bulk phase of the compound concentration.

The values of two parameters (see Table 2), defined as the maximum surface excess concentration (Γ_max_) and the area occupied by a single compound molecule at the air−water interface (A_min_), have been compiled [2,38]. In the literature describing the adsorption of compound molecules at the air–water interface, the values of the aforementioned parameters are inherently linked to this phenomenon and often exhibit a specific trend, namely, as the value of Γ_max_ increases, the value of A_min_ decreases [43]. This trend can be observed by focusing on the results for the A_min_ and Γ_max_ values obtained for the synthesized monocationic and dicationic SAILs. According to Wang et al. [56], this situation (higher Γ_max_ and simultaneously lower A_min_) results in more densely and tightly packed molecules of surface-active compounds at the interface. Additionally, based on the theory of hydrophobicity, the ability of compound molecules to move toward the air–water interface changes with the length of the alkyl chain [57].

The ΔG^0^_ads_ values listed in Table 2 are negative for both monocationic and dicationic SAILs, indicating that the adsorption process occurs spontaneously. Furthermore, data in the literature suggest that ΔG^0^_ads_ values tend to become more negative as the hydrocarbon chain length increases [50,58]. Perinelli et al. [59] demonstrated that the increased hydrophobicity of amphiphilic molecules enhances their ability to adsorb and self-aggregate at the air–water interface. However, this trend was not observed for the synthesized compounds. It is possible that these molecules arrange themselves in a manner that promotes the spontaneity of the adsorption process at the liquid–water interface, resulting in lower parameter values. Similarly, Banjare et al. [60] emphasized that the amphiphilic segment of a surface-active compound significantly influences surface behavior, ultimately shaping adsorption dynamics.

**Figure 5:**
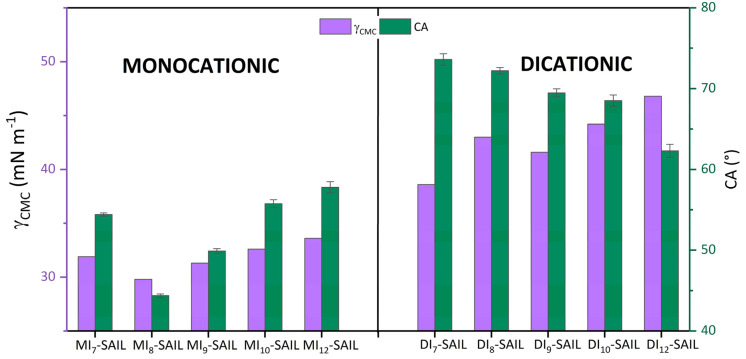
The left *Y*-axis shows the surface tension at CMC (γ_CMC_) vs. the effect of elongation of the hydrophobic alkyl part of SAILs. The right *Y*-axis presents the contact angle (CA) values vs. the same function of SAILs.

Focusing on the discussion of the wettability of the synthesized SAILs, it is necessary to cite the ranges of CA values by which the compounds are classified. Accordingly, CA values in the range from 0° to 90° refer to compounds that partially wet the surface under study. For monocationic SAILs (from 44.38° to 57.79°) and dicationic SAILs (from 62.31° to 73.61°), the CA value falls within the indicated range. Continuing with the interpretation of the degree of wettability of the compounds, Figure 5 compares the values for CA with those for the parameter γ_CMC_.

In the earlier part of this work, the γ_CMC_ and CA values were discussed in detail. However, when looking through the lens of these two parameters related to the surface activity of the studied compounds, an interesting relationship can be observed. As we mentioned, the lowest γ_CMC_ value was obtained for **MI_8_-SAIL**, which thus best wetted the hydrophobic surface. Conversely, the compound that lowered the surface tension at CMC also wetted the analyzed surface the least. This trend holds true for monocationic SAILs, but not for dicationic SAILs. This may be related to the promotion of steric hindrance between SAIL molecules, which could be caused by the way these compound molecules form in aqueous solution [50,61,62].

### 2.4. Atomic Force Microscopy (AFM) Analysis

**Figure 6:**
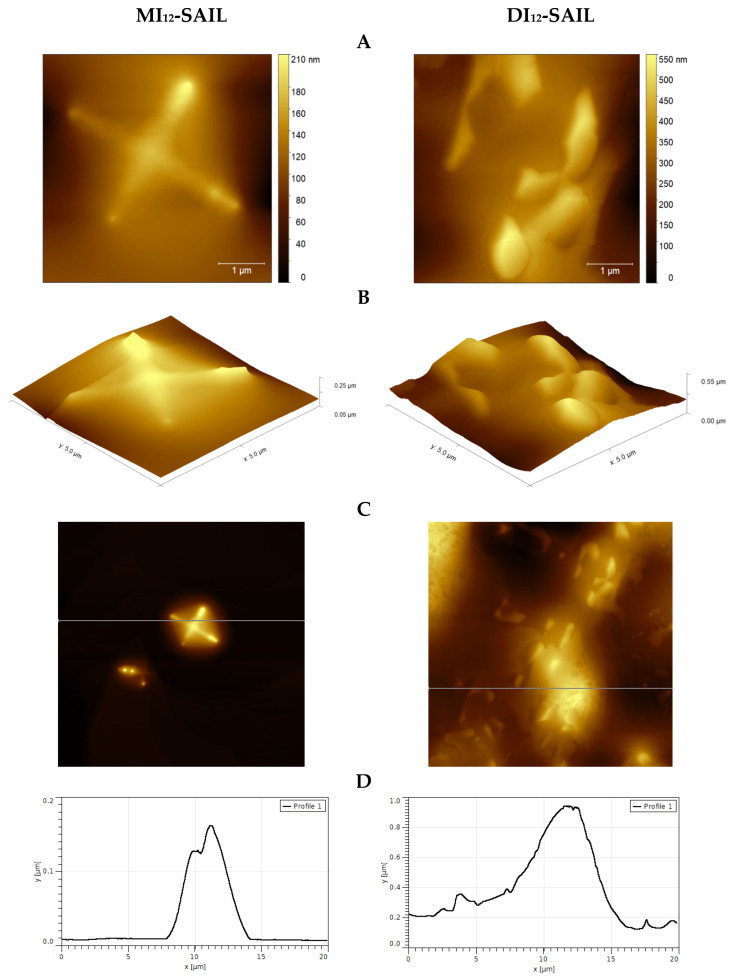
AFM studies of monocationic and dicationic SAILs deposited on a mica surface (**A**–**D**); topography of selected areas of samples (**A**,**C**); 3D view of the test surfaces (**B**); profile curves for selected deposits (**D**).

Given the potential use of the analyzed compounds, we used the (AFM) technique to perform a topographical analysis of the model hydrophobic mica surfaces. The selection of compounds for the study (**MI_12_-SAIL** and **DI_12_-SAIL**) was driven by the highest surface activity. Figure 6 presents exemplary images from the results of the AFM studies.

Looking at the results, it is important to focus on several aspects, particularly the difference between monocationic and dicationic SAILs in the way they cover the mica surface. Generally, a layered coverage of the examined surface can be observed. This may be due to the concentration of the compounds used, which was above the CMC. As a result, SAIL molecules tended to form layered structures. Moreover, in the case of **MI_12_-SAIL**, clusters can be observed, which are significantly different from those observed for **DI_12_-SAIL**. This is because, for the compound with a double amphiphilic part, the clusters form more spherical aggregates than in the case of monocationic SAIL. According to our interpretation, the topography of the study area (see Figure 6A–D) covered with **MI_12_-SAIL** and **DI_12_-SAIL** shows a dense distribution of deposits on the surface, which seem to have a round shape. The mean deposit sizes were around 4.3 μm (**MI_12_-SAIL**) and 5.5 μm (**DI_12_-SAIL**), which confirm the abovementioned conclusion. Moreover, in the case of **DI_12_-SAIL**, a denser distribution of deposits on the surface can be observed, which is likely due to the higher surface activity of this compound and, therefore, more effective surface coverage.

### 2.5. Antimicrobial Properties

The antimicrobial activity of the ten imidazolium-based surface-active ionic liquids (SAILs) was assessed by their minimum inhibitory concentration (MIC) values, reported in mmol L^−1^, against various microorganisms, including Gram-positive bacteria (*S. aureus* [SAU] and *E. faecalis* [EFA]), Gram-negative bacteria (*P. aeruginosa* [PAE], *E. coli* [ECO], and *K. pneumoniae* [KPN]), and fungi (*C. albicans* [CAL]). Lower MIC values indicate higher antimicrobial efficacy (see Table 3).

Among the monocationic SAILs, **MI_7_-SAIL** displayed moderate antimicrobial activity, particularly against *S. aureus* (0.114 mmol L^−1^) and *E. faecalis* (0.228 mmol L^−1^), though its effectiveness against Gram-negative bacteria, especially *P. aeruginosa* (3.562 mmol L^−1^), was notably weaker. **MI_8_-SAIL**, on the other hand, showed strong antimicrobial potency across most strains, with low MIC values against *S. aureus* (0.014 mmol L^−1^), *E. faecalis* (0.027 mmol L^−1^), and *C. albicans* (0.027 mmol L^−1^), although its activity against Gram-negative bacteria was moderate. **MI_9_-SAIL** also exhibited good activity, particularly against *S. aureus* and *E. faecalis* (both 0.026 mmol L^−1^), but was less effective against *P. aeruginosa* (1.636 mmol L^−1^), though more potent against *K. pneumoniae* (0.422 mmol L^−1^) and *C. albicans* (0.053 mmol L^−1^). **MI_10_-SAIL** emerged as one of the most effective compounds, showing low MIC values against all tested strains, including *S. aureus* (0.013 mmol L^−1^), *E. faecalis* (0.013 mmol L^−1^), and *C. albicans* (0.025 mmol L^−1^), with only slightly reduced activity against *P. aeruginosa*. **MI_12_-SAIL**, while highly effective against *S. aureus* (0.012 mmol L^−1^) and *E. faecalis* (0.024 mmol L^−1^), demonstrated weaker activity against P. *aeruginosa* (5.937 mmol L^−1^) but maintained moderate potency against other strains, including E. coli, K. pneumoniae, and *C. albicans*.

The dicationic SAILs generally exhibited stronger antimicrobial properties. **DI_7_-SAIL** showed good efficacy against Gram-positive bacteria (*S. aureus* and *E. faecalis*: 0.066 mmol L^−1^) but weaker activity against *P. aeruginosa*. **DI_8_-SAIL** demonstrated a similar performance, with good activity against Gram-positive bacteria and fungi, but limited efficacy against Gram-negative bacteria. **DI_9_-SAIL** displayed more balanced activity across both Gram-positive and Gram-negative strains, with low MIC values for *S. aureus* (0.016 mmol L^−1^) and moderate effectiveness against *P. aeruginosa*, *E. coli*, and *K. pneumoniae*. **DI_10_-SAIL** showed moderate to strong antimicrobial activity, though it had a relatively high MIC for *S. aureus* (0.482 mmol L^−1^) compared to the other dicationic SAILs. **DI_12_-SAIL** was the most potent, showing low MIC values across all strains, including *S. aureus* (0.015 mmol L^−1^), *P. aeruginosa* (0.923 mmol L^−1^), and *C. albicans* (0.007 mmol L^−1^).

Compared to the control compounds, **DDAC** and **BAC**, which showed excellent antimicrobial activity, particularly against *S. aureus* (0.004 mmol L^−1^) and fungi (*C. albicans*: 0.007 mmol L^−1^), most SAILs performed competitively. The dicationic SAILs, particularly **DI_12_-SAIL**, emerged as the most promising candidates for broad-spectrum antimicrobial activity, rivaling the control agents in potency.

The antimicrobial efficacy of imidazolium-based SAILs, especially when comparing monocationic and dicationic compounds, has been well-documented in the literature [18,59]. Dicationic SAILs consistently demonstrate superior antimicrobial activity compared to their monocationic counterparts, as observed in the data from various studies [2,38].

One primary reason for the heightened activity of dicationic SAILs is their increased charge density. Studies have shown that dicationic SAILs possess stronger electrostatic interactions with the negatively charged microbial membranes, including the anionic phospholipids and lipopolysaccharides present in Gram-negative bacteria. For example, in a 2015 study by Singh et al. [63], dicationic SAILs exhibited significantly lower MIC values against *E. coli* and *P. aeruginosa* compared to monocationic SAILs, largely due to their ability to better penetrate and destabilize bacterial membranes. Similar findings were reported by Zhao et al. [64], where dicationic SAILs demonstrated a 2–3-fold reduction in MIC values against Gram-positive bacteria, including *S. aureus* and *E. faecalis*.

The dicationic compound **DI_12_-SAIL** exhibited MIC values as low as 0.015 mmol L^−1^ against both *S. aureus* and *E. coli*, highlighting its broad-spectrum antimicrobial potential. In contrast, **MI_7_-SAIL**, a monocationic counterpart, showed significantly higher MIC values of 0.114 mmol L^−1^ and 0.228 mmol L^−1^ against the same strains, reinforcing the literature’s findings that charge density plays a pivotal role in membrane disruption efficacy.

The enhanced flexibility provided by the ether linkage in dicationic SAILs is another factor contributing to their improved antimicrobial performance. Research by Pham-Truong et al. [65] emphasized that SAILs with more flexible spacers between imidazolium groups allow the molecules to better conform to microbial membranes, facilitating deeper penetration and increased disruption of lipid bilayers. This flexibility is particularly advantageous when targeting organisms with complex membrane structures, such as *P. aeruginosa*, a Gram-negative bacterium known for its robust outer membrane. The MIC values for **DI_12_-SAIL** against *P. aeruginosa* (0.923 mmol L^−1^) were markedly lower compared to monocationic **MI_12_-SAIL** (5.937 mmol L^−1^), supporting this notion.

A key advantage of dicationic SAILs, as highlighted in the literature, is their broad-spectrum antimicrobial activity. Dicationic SAILs, due to their enhanced charge density and structural flexibility, have been shown to be effective against a wide range of bacteria and fungi. A study by Moshikur et al. [66] demonstrated that dicationic SAILs could inhibit both Gram-positive and Gram-negative bacteria at lower concentrations compared to monocationic counterparts. Additionally, their antifungal activity was particularly noteworthy. In our study, **DI_12_-SAIL** exhibited an impressive MIC of 0.007 mmol L^−1^ against *C. albicans*, which aligns with similar findings in the literature showing that dicationic SAILs outperformed conventional antifungal agents, such as amphotericin B and fluconazole, at equivalent concentrations.

Although dicationic SAILs demonstrate greater antimicrobial potency, several studies, including one by Zhang et al. [67], have raised concerns about their potential cytotoxicity toward human cells at higher concentrations due to the very same membrane-disrupting properties that make them effective antimicrobials. However, these concerns are often mitigated by optimizing the alkyl chain length and functional groups, as seen with **DI_12_-SAIL**, which strikes a balance between antimicrobial potency and reduced toxicity, making it a promising candidate for further development.

When compared to standard quaternary ammonium compounds (QACs) such as **DDAC** and **BAC**, dicationic SAILs show comparable, if not superior, activity. **DI_12_-SAIL** performed on par with **DDAC**, showing similar MIC values against *S. aureus* (0.015 mmol L^−1^ for **DI_12_-SAIL** vs. 0.004 mmol L^−1^ for **DDAC**). However, **DI_12_-SAIL** had a broader activity spectrum, particularly against Gram-negative bacteria like *E. coli* and *P. aeruginosa*, where QACs typically show reduced effectiveness due to their inability to penetrate the outer membrane. This positions dicationic SAILs as versatile alternatives or adjuncts to conventional disinfectants and antiseptics.

The scientific literature consistently supports the superior antimicrobial efficacy of dicationic SAILs over monocationic ones. Factors such as increased charge density, stronger electrostatic interactions with microbial membranes, structural flexibility due to ether linkages, and broad-spectrum activity contribute to the lower MIC values and greater potency of dicationic SAILs. This aligns well with the provided data [17], which show that **DI_12_-SAIL**, in particular, demonstrated strong antimicrobial and antifungal activity. While potential cytotoxicity and resistance issues warrant further study, dicationic SAILs represent promising candidates for broad-spectrum antimicrobial agents, with potential applications in both medical and industrial settings.

**Figure 7:**
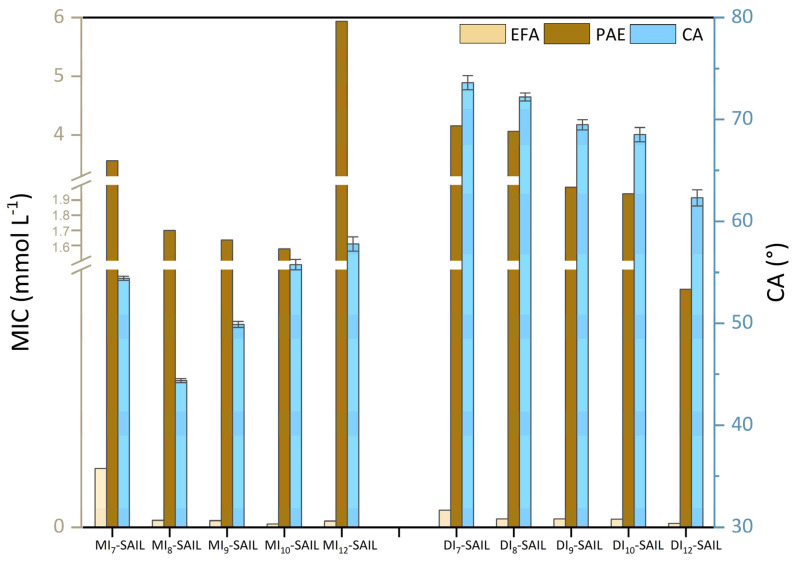
Correlation of biological activity (MIC values) with surface activity (CA values).

Based on the results presented in Table 2 and Table 3, it was decided to compare biological activity with surface properties. For this purpose, for selected representatives of G-positive bacteria (*E. faecalis* [EFA]) and G-negative bacteria (*P. aeruginosa* [PAE]), the CA values were correlated with MIC values (see Figure 7). However, for fungal species, the correlation could not be clearly determined.

To ensure clarity in the analysis of the results, we structured our discussion according to the size of the amphiphilic part in the SAILs’ structures. Interestingly, in the case of monocationic SAILs, a similar parabolic tendency was observed. According to microbiological studies, this phenomenon is associated with the cut-off effect [68]. Nevertheless, the lowest CA value was obtained for **MI_8_-SAIL**, while the lowest MIC value was recorded for **MI_10_-SAIL**. Although it is not possible to distinctly identify a single potentially best-performing compound among all monocationic SAILs as part of a commercial surface-active formulation with biocidal action, this does not change the fact that the synthesized SAILs represent an interesting group, and research on them should be continued in the future. In contrast, for dicationic SAILs, a different trend was observed. In their case, as the degree of wettability increases, the antimicrobial activity of the compounds also increases, but only for bacterial species. Vallapa et al. [69] highlighted a similar tendency in their analysis of biological materials. Their speculation is important from an application point of view, as the larger the range of action of the compound, the more effective the product containing the given SAIL becomes. 

## 3. Materials and Methods

### 3.1. Thermal Analysis

Thermal characterization of the synthesized ionic liquids was performed using two methods, i.e., differential scanning calorimetry (DSC) and thermogravimetric analysis (TG).

Phase transition temperatures of the obtained ionic liquids were determined using the DSC method. For this purpose, a sample of 5–10 mg of SAIL was weighed into a DSC crucible. Then, the crucible was closed with a lid containing a hole to allow the escape of gases generated during the heating process. The crucible prepared in this way was placed in the chamber of the DSC 1 apparatus (Mettler-Toledo, Greifensee, Switzerland). The sample was heated/cooled at a rate of 10 °C min^−1^ in the temperature range from −70 °C to 120 °C, in an inert gas–argon atmosphere, which flowed through the chamber at a rate of 50 mL min^−1^. The temperatures of characteristic phase transitions, such as cold crystallization (or recrystallization, T_cc_), glass transition (T_g_), and melting (T_m_) temperatures, were determined from the second heating cycle of the DSC thermograms. The crystallization temperature (T_c_) was determined from the cooling cycle of the DSC thermogram.

The thermal stability of the synthesized compounds was tested by the TG method using the Tarsus analyzer instrument TG 209 F3 (NETZSCH-Geratebau GmbH, Selb, Germany). The synthesized compound was weighed in a platinum crucible in the amount of 10 ± 0.5 mg, then placed in the apparatus chamber and heated at a rate of 10 °C min^−1^ in the temperature range of 35–600 °C. Inert gas, i.e., nitrogen, flowed through the chamber at a rate of 30 mL min^−1^ (protective gas flow: 10 mL min^−1^; purge gas flow: 20 mL min^−1^).

### 3.2. Surface Activity

Surface activity measurements were primarily performed using a DSA 100 analyzer (Krüss, Hamburg, Germany) with an accuracy of ±0.01 mN m^−1^ and a Fisherbrand FBH604 thermostatic bath (Fisher, Schwert, Germany) with an accuracy of ±0.1 °C to keep fire conditions constant. The measurement of surface tension (γ in mN m^−1^) based on the Shape Drop Method was conducted by analyzing the drop profile using the mathematical Laplace equation. The wettability was assessed based on the measurement of the contact angle (CA), conducted according to the following procedure. Initially, a droplet was deposited onto a hydrophobic surface (paraffin). Once the drop shape and contact line were identified, the shape was fitted to a mathematical model based on the Young–Laplace equation. All measurements were digitized by a CCD camera.

Based on the measurements performed, the values of parameters such as the critical micelle concentration (CMC), the surface tension at the CMC (γ_CMC_), the Gibbs free energy of adsorption (ΔG^0^_ads_), the adsorption efficiency (pC_20_), the surface excess concentrations at the saturated interface (Γ_max_), and the minimum surface occupied by a molecule at the interface (A_min_) were calculated. Moreover, the values of the following parameters were determined based on the provided equations [2,40,41]:

Gibbs free energy of adsorption:

$${{∆G}^{0}}_{ads}=-RTlna$$

where

R is the gas constant;
T is the absolute temperature.

The a value is a parameter of the Szyszkowski equation:

$$\gamma =\gamma_{0}[1-bln\left(\frac{c}{a}+1\right)]$$

where

γ_0_ is the surface tension of the pure water;
b and a are empirical constants;
c is the SAIL concentration.

Adsorption efficiency:

$${pC}_{20}=-\log C_{20}$$

Surface excess concentrations (Γ_max_) were calculated from the slope of the linear portion of the γ-log C plots (Figure 4) using the Gibbs isotherm:

$$\Gamma_{max}=-\frac{1}{RT}\cdot \frac{d\gamma }{d(\ln C)}$$

where

R is the gas constant;
T is the absolute temperature;
C is the concentration of salts.

From Γ_max_, the minimum surface occupied by a molecule at the interface (A_min_) can be calculated using the following equation:

$$A_{min}=\frac{1}{\Gamma_{max}N_{A}}$$

where

N_A_ is the Avogadro number.

### 3.3. Atomic Force Microscopy (AFM)

The samples of each SAIL were dissolved in a water. After that, the solutions of the samples were applied to a freshly cleaned piece of mica (1 × 1 cm) using a syringe. This mica was previously adhered to a steel disc with double-sided tape. The prepared samples were left to air-dry for 24 h under conditions that minimized the risk of airborne contamination. The study was conducted using an NX10 microscope obtained from Park Systems Mannheim, Germany, and images were captured in non-contact mode. The microbeams utilized for the measurements were All-In-One D models manufactured by Budgetsensors, Sofia, Bulgaria featuring a nominal elastic constant of 40 N m^−1^. The tests were carried out at constant room temperature (23 °C). Each image had a resolution of 512 × 512 pixels, with a scan speed ranging from 0.3 to 0.5 Hz. For every sample, images at 15 × 15-, 5 × 5-, 2 × 2-, and 0.5 × 0.5-micrometer scales were obtained. The images were subsequently analyzed with Gwyddion software, version 2.67.

### 3.4. Antimicrobial Activity

Microbial Strains

The antimicrobial properties of the synthesized compounds were assessed against a diverse range of microbial strains, including *Staphylococcus aureus*, *S. aureus* (SAU) ATCC 25213, *Pseudomonas aeruginosa*, *P. aeruginosa* (PAE) ATCC 27853, *Klebsiella pneumoniae*, *K. pneumoniae* (KPN) ATCC 700603, *Escherichia coli*, *E. coli* (ECO) ATCC 25922, E*nterococcus faecalis*, *E. faecalis* (EFA) ATCC 29212, and *Candida albicans* (*C. albicans* (CAL) ATCC 90028). These reference strains were obtained from Microbiologics (San Diego, CA, USA). Prior to testing, all microbial cultures were stored at −80 °C in cryovial vials (Simport, Bernard-Pilon Beloeil, Quebec, Canada) and subcultured in Müller–Hinton broth to ensure optimal growth and viability during the experiments.

Determination of Minimum Inhibitory Concentration (MIC)

To evaluate the antimicrobial efficacy of the synthesized compounds, minimum inhibitory concentration (MIC) values were determined for several clinically relevant microbial strains. The microbial strains, sourced from Microbiologics (San Diego, CA, USA), were prepared for testing by standardizing bacterial and fungal suspensions to a turbidity equivalent to 0.5 McFarland standards. This was achieved by collecting growth from blood trypticase soy agar (TSA and BBL) plates and suspending the cells in 2 mL of sterile saline.

MIC values were determined by preparing a series of agar plates containing decreasing concentrations of the tested compounds, ranging from 1.0% to 0.000125% (*w*/*v*). Bacteria from standardized cultures were inoculated onto these plates, and after incubation, bacterial growth was assessed. The MIC was defined as the lowest concentration of the compound at which no more than one bacterial colony developed, indicating effective inhibition of microbial growth. Two types of controls were utilized during the experiment: a growth control and a sterile control. The growth control contained bacterial suspensions without any antimicrobial agents, allowing for the natural progression of bacterial growth, while the sterile control consisted solely of growth medium without bacteria or antimicrobial compounds. The sterile control served as a negative baseline, ensuring that no bacterial contamination occurred during the procedure.

Mueller–Hinton agar (bio-Merieux, Lombard, IL, USA) was employed as the test medium for bacterial cultures. The MIC was defined as the lowest concentration of the compound at which no visible bacterial growth was observed, apart from a barely noticeable haze. Readings were taken after 24 or 48 h of incubation at 37 °C. The overall antimicrobial testing procedure was conducted in strict accordance with the Clinical and Laboratory Standards Institute (CLSI) guidelines to ensure accuracy and reliability in assessing the microbial susceptibility of the compounds under investigation [70].

## 4. Conclusions

This study adopted a compelling strategy to explore the imidazolium group of ionic liquids, with surface activity taking center stage. Monocationic and dicationic imidazolium compounds with surface activity were synthesized in high yields (≥90%), and their structures were confirmed by ^1^H NMR and ^13^C NMR. By examining the melting points, we categorized the salts studied into ionic liquid groups. Owing to their amphiphilic characteristics, monocationic and dicationic ILs are surface-active compounds, a term that appropriately describes surface-active ionic liquids (SAILs). Additionally, the surface activity results showed that the studied monocationic and dicationic imidazolium SAILs effectively reduced the surface tension of water. For monocationic SAILs, this reduction ranged from 29.8 to 33.6 mN m^−1^, which was lower than that achieved by the cationic surfactant **BAC** and close to that of **DDAC** [45]. Meanwhile, for dicationic SAILs, the surface tension ranged from 41.6 to 47.4 mN m^−1^, a range that includes the γ_CMC_ value for **C_12_TAB**. Through a detailed study of CMC values, which are considered indicators of a compound’s surface activity, we found that introducing a hydrophobic chain of the same length as that in conventional surfactants results in higher surface activity. Consequently, the newly synthesized compounds were classified as unique compared to structurally analogous surfactants. Topography analyses showed that the coverage of the model surface depends on the amount of amphiphilic component in the cation. In conclusion, both monocationic and dicationic SAILs with hydrophobic chains or spacers of up to twelve carbon atoms have been shown to be highly promising surfactants. Conversely, when seeking the most effective disinfectant that also functions as a surface-active agent, it is easier to identify a suitable compound among the dicationic SAILs, particularly those with 12 carbon atoms in the spacer. Notably, **DI_12_-SAIL** exhibited an impressive MIC of 0.007 mmol L^−1^ against *C. albicans*, consistent with findings in the literature showing that dicationic SAILs outperformed conventional antifungal agents, such as amphotericin B and fluconazole, at equivalent concentrations. In the case of monocationic SAILs, the most effective compounds are those with alkyl chains longer than nine carbon atoms.

The key takeaway is that, despite the many structural variations in imidazolium SAILs documented in the literature, the entire series of compounds studied reveal a diverse range of potential applications, highlighting their significant promise for beneficial uses. As a result, these findings could open up new opportunities to advance sustainable chemistry by introducing a set of alternative compounds that may be more effective than conventional cationic surfactants.

## Figures and Tables

**Figure 1:**
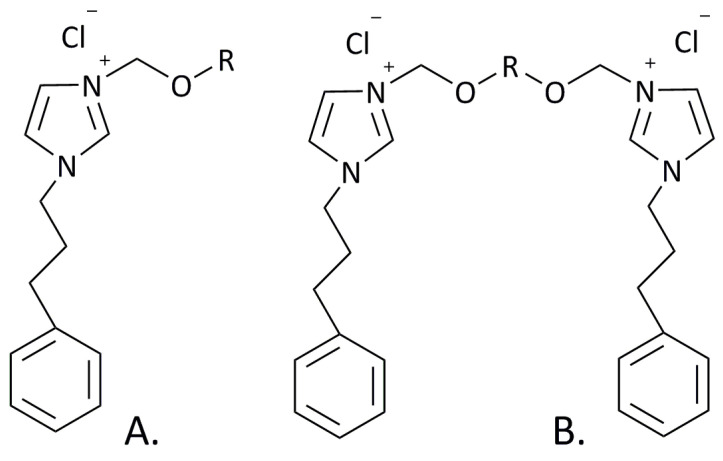
Molecular structures of synthetized monocationic (**A**) SAILs, where R = C_7_H_15_, C_8_H_17_, C_9_H_19_, C_10_H_21_, or C_12_H_25_, and dicationic (**B**) SAILs, where R = C_7_H_14_, C_8_H_16_, C_9_H_18_, C_10_H_20_, or C_12_H_24_.

**Table 1:** Thermal properties of a series of imidazolium-based SAILs (T_5%_—the temperature of loss of 5% of sample mass, T_10%_—the temperature of loss of 10% of sample mass, T_50%_—the temperature of loss of 50% of sample mass, T_90%_—the temperature of loss of 90% of sample mass, R—residual mass, T_g_—glass transition temperature, T_cc_—cold crystallization temperature, T_m_—melting temperature, T_c_—crystallization temperature).

	TGA						DSC		
Abbreviation	T_5%_ (°C)	T_10%_ (°C)	T_50%_ (°C)	T_90%_ (°C)	R (%)	T_g_ (°C)	T_cc_ (°C)	T_m_ (°C)	T_c_ (°C)
**MONOCATIONIC SAILs**									
**MI_7_-SAIL**	124.1	149.8	238.6	270.5	1.0	-	-	−13.1	−33.3
**MI_8_-SAIL**	143.2	176.8	235.1	270.3	1.8	−19	-	19.8	−1.0/−2.3/−8.5/−22.9/−46.3
**MI_9_-SAIL**	143.0	170.2	239.3	270.3	1.7	−28.8	-	9.1	−15.1/−25.0
**MI_10_-SAIL**	152.2	178.3	236.0	275.8	1.9	−35.2	−13.3	−1.4/7.6/19.4	−4.3/−6.0/−8.4/−11.0/−20.0
**MI_12_-SAIL**	182.0	204.2	246.8	290.1	2.8	−26.7	−11.0	9.2/20.6/31.0	9.6/−11.0
**DICATIONIC SAILs**									
**DI_7_-SAIL**	179.8	210.4	264.1	447.7	9.0	−42.5	-	-	-
**DI_8_-SAIL**	198.2	220.0	275.0	439.2	7.6	−40.2	-	-	-
**DI_9_-SAIL**	186.2	212.0	281.1	450.4	9.2	−39.4	7.3	37.5/50.9	-
**DI_10_-SAIL**	192.9	218.7	278.0	415.8	5.6	−35.3	-	18.2/31.2/46.8/57.8	18.6
**DI_12_-SAIL**	192.1	215.9	284.0	428.1	5.3	−29.0	-	16.3/41.3/63.2/70.9	37.6/6.4

**Table 2:** Surface properties of a series of imidazolium SAILs.

Abbreviation	CMC (mmol L^−1^)	pC_20_	γ_CMC_ (mN m^−1^)	CA (°)	Γ_max_ × 10^6^ (mol m^−2^)	A_min_ × 10^19^ (m^2^)	ΔG^0^_ads_ (kJ m^−1^)
**MONOCATIONIC SAILs**							
**MI_7_-SAIL**	38.54	2.63	31.9	54.42	4.28	3.88	−15.2
**MI_8_-SAIL**	6.77	3.29	29.8	44.38	3.64	4.56	−23.9
**MI_9_-SAIL**	6.13	3.06	31.3	49.89	4.34	3.83	−21.6
**MI_10_-SAIL**	3.03	3.17	32.6	55.75	4.72	3.53	−22.2
**MI_12_-SAIL**	1.32	3.79	33.6	57.79	3.64	4.56	−26.8
**DICATIONIC SAILs**							
**DI_7_-SAIL**	47.35	2.28	38.6	73.61	2.41	6.89	−24.5
**DI_8_-SAIL**	12.06	2.37	43.0	72.21	3.15	5.27	−19.0
**DI_9_-SAIL**	8.44	2.89	41.6	69.48	2.75	6.05	−20.1
**DI_10_-SAIL**	0.85	3.54	44.2	68.52	3.99	4.16	−26.4
**DI_12_-SAIL**	0.28	3.87	46.8	62.31	4.53	3.66	−23.7
**BAC**	4.29 ^a^	3.23 ^a^	36.5 ^a^	67.90 ^a^			−27.03 ^a^
**DDAC**	1.69~2.00 ^b^	4.13 ^a^	27.1 ^a^	41.03 ^a^			−32.31 ^a^
**DomphB**	1.78 ^c^		36.5 ^c^				
**C_10_TAB**	67.00 ^d^		40.0 ^d^				
**C_12_TAB**	15.00 ^d^		33.2–39.0 ^d^				

**BAC**—benzalkonium chloride, **DDAC**—didecyldimethylammonium chloride, **DomphB**—domiphen bromide, **C_10_TAB**—decyltrimethylammonium bromide, **C_12_TAB**—dodecyltrimethylammonium bromide. Accuracy of contact angle (CA) values: ± 0.6°. ^a^ From [40]. ^b^ From [45]. ^c^ From [46]. ^d^ From [47]. All values were obtained from surface tension measurements.

**Table 3:** Summary of MIC values for selected G-positive bacteria and G-negative bacteria and fungal species.

MIC (mmol L^−1^)						
Abbreviation	G(+) Bacteria		G(−) Bacteria			Fungi
	*S. aureus*	*E. faecalis*	*P. aeruginosa*	*E. coli*	*K. pneumoniae*	*C. albicans*
**MONOCATIONIC SAILs**						
**MI_7_-SAIL**	0.114	0.228	3.562	0.228	1.767	0.228
**MI_8_-SAIL**	0.014	0.027	1.699	0.110	1.699	0.027
**MI_9_-SAIL**	0.026	0.026	1.636	0.106	0.422	0.053
**MI_10_-SAIL**	0.013	0.013	1.578	0.051	0.789	0.025
**MI_12_-SAIL**	0.012	0.024	5.937	0.380	2.969	0.024
**DICATIONIC SAILs**						
**DI_7_-SAIL**	0.066	0.066	4.155	0.066	1.030	0.017
**DI_8_-SAIL**	0.032	0.032	4.061	0.032	4.061	0.016
**DI_9_-SAIL**	0.016	0.032	1.985	0.032	0.985	0.016
**DI_10_-SAIL**	0.482	0.031	1.942	0.016	0.963	0.016
**DI_12_-SAIL**	0.015	0.015	0.923	0.015	0.461	0.007
**BAC**	0.028	0.014	1.765	0.057	0.456	0.014
**DDAC**	0.004	0.007	0.856	0.014	0.856	0.007

## Data Availability

Data generated and analyzed during this study are included in this manuscript and in the Supplementary Materials.

## Supplementary Materials

The following supporting information can be downloaded at: https://www.mdpi.com/article/10.3390/molecules29235743/s1, Figure S1. DSC thermograms of synthetized SAIL (Black line—heating segment, Red line—cooling segment). Figure S2. Relationship between surface tension (γ) and the logarithm of the concentration of synthetized compounds (log C), monocationic SAILs (left side), and dicationic SAILs (right side). Figure S3. A monocationic SAIL, where x = 4 (for R = C_7_H_15_), 5 (C_8_H_17_), 6 (C_9_H_19_), 7 (C_10_H_21_), and 9 (C_12_H_25_), with numbered hydrogen atoms. Figure S4. B dicationic SAIL, where x = 3 (for R = C_7_H_14_), 4 (C_8_H_16_), 5 (C_9_H_18_), 6 (C_10_H_20_), and 8 (C_12_H_24_), with numbered hydrogen atoms. Figure S5. ^1^H NMR spectrum of the monocationic SAIL, where R = C_7_H_15_. Figure S6. ^13^C NMR spectrum of the monocationic SAIL, where R = C_7_H_15_. Figure S7. ^1^H NMR spectrum of the monocationic SAIL, where R = C_8_H_17_. Figure S8. ^13^C NMR spectrum of the monocationic SAIL, where R = C_8_H_17_. Figure S9. ^1^H NMR spectrum of the monocationic SAIL, where R = C_9_H_19_. Figure S10. ^13^C NMR spectrum of the monocationic SAIL, where R = C_9_H_19_. Figure S11. ^1^H NMR spectrum of the monocationic SAIL, where R = C_10_H_21_. Figure S12. ^13^C NMR spectrum of the monocationic SAIL, where R = C_10_H_21_. Figure S13. ^1^H NMR spectrum of the monocationic SAIL, where R = C_12_H_25_. Figure S14. ^13^C NMR spectrum of the monocationic SAIL, where R = C_12_H_25_. Figure S15. ^1^H NMR spectrum of the dicationic SAIL, where R = C_7_H_14_. Figure S16. ^13^C NMR spectrum of the dicationic SAIL, where R = C_7_H_14_. Figure S17. ^1^H NMR spectrum of the dicationic SAIL, where R = C_8_H_16_. Figure S18. ^13^C NMR spectrum of the dicationic SAIL, where R = C_8_H_16_. Figure S19. ^1^H NMR spectrum of the dicationic SAIL, where R = C_9_H_18_. Figure S20. ^13^C NMR spectrum of the dicationic SAIL, where R = C_9_H_18_. Figure S21. ^1^H NMR spectrum of the dicationic SAIL, where R = C_10_H_20_. Figure S22. ^13^C NMR spectrum of the dicationic SAIL, where R = C_10_H_20_. Figure S23. ^1^H NMR spectrum of the dicationic SAIL, where R = C_12_H_24_. Figure S24**.** ^13^C NMR spectrum of the dicationic SAIL, where R = C_12_H_24_.
