# Supplementary material for: Impact of Alkyl Spacer and Side Chain on Antimicrobial Activity of Monocationic and Dicationic Imidazolium Surface-Active Ionic Liquids: Experimental and Theoretical Insights
Source: Molecules. 2024 Dec 5;29(23):5743. doi: 10.3390/molecules29235743 (PMC11643617; doi:10.3390/molecules29235743)
Supplement: Supplementary file 1 [file molecules-29-05743-s001.zip › molecules-3342019-supplementary.pdf]

Impact of Alkyl Spacer and Side Chain on Antimicrobial Activity of Monocationic and Dicationic Imidazolium Surface-Active Ionic Liquids: Experimental and Theoretical Insights

Marta Wojcieszak [1], Sylwia Zięba [2], Alina T. Dubis [3], Maciej Karolak [4], Łukasz Pałkowski [4], Agnieszka Marcinkowska [1], Andrzej Skrzypczak [1†], Alicja Putowska[1] and Katarzyna Materna[1,*]

[1] Faculty of Chemical Technology, Poznan University of Technology, Berdychowo 4, 60-965 Poznań, Poland; marta.wojcieszak@put.poznan.pl (M.W.); agnieszka.marcinkowska@put.poznan.pl (A.M.); alicja.putowska@student.put.poznan.pl (A.P.)

[2] Department of Molecular Crystals, Institute of Molecular Physics Polish Academy of Sciences, Smoluchowskiego 17, 60-179 Poznań, Poland; sylwia.zieba@ifmpan.poznan.pl

[3] Faculty of Chemistry, University of Bialystok, Ciołkowskiego 1K, 15-245 Białystok, Poland; alina@uwb.edu.pl

[4] Department of Pharmaceutical Technology, Faculty of Pharmacy, Nicolaus Copernicus University, Jurasza 2, 85-089 Bydgoszcz, Poland; maciej.karolak@cm.umk.pl (M.K.); lukaszpalkowski@cm.umk.pl (Ł.P.)

* Correspondence: katarzyna.materna@put.poznan.pl

† Deceased September 2023. Dedicated to the memory of dr Andrzej Skrzypczak

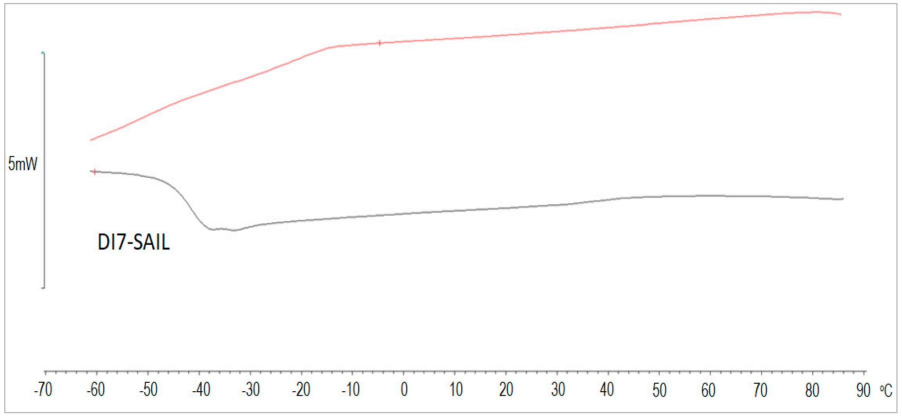

DI$_7$-SAIL

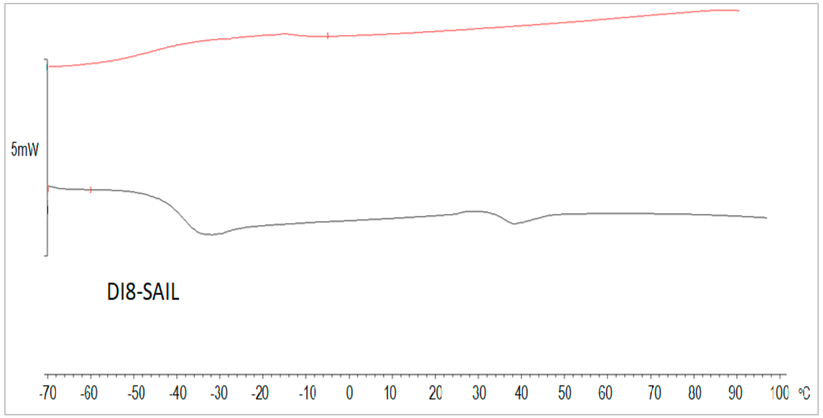

DI$_8$-SAIL

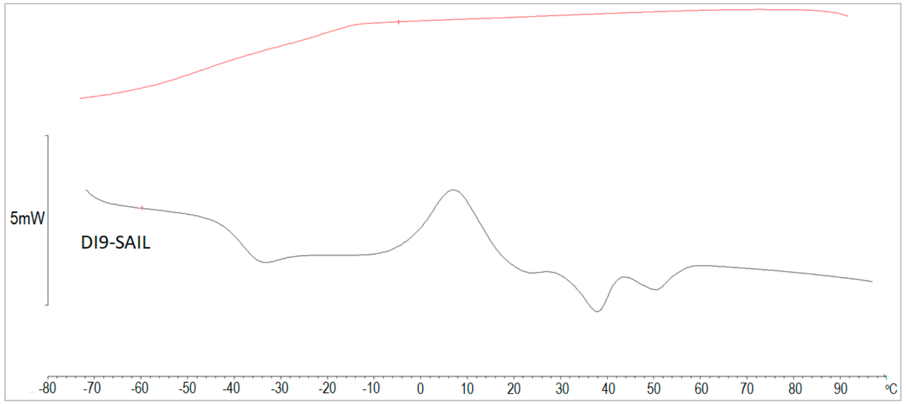

DI$_9$-SAIL

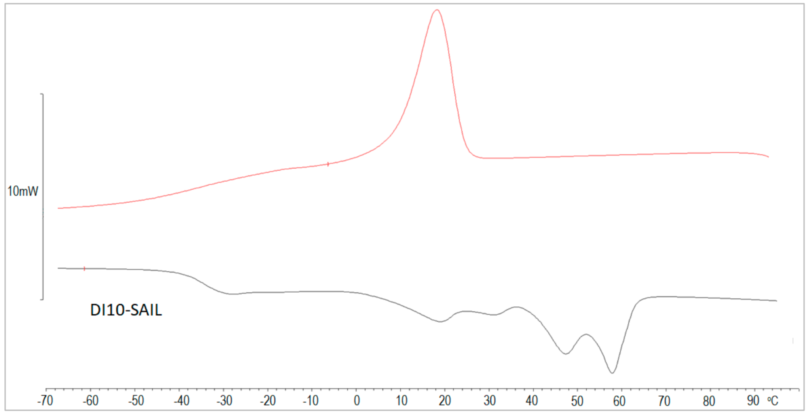

DI$_{10}$-SAIL

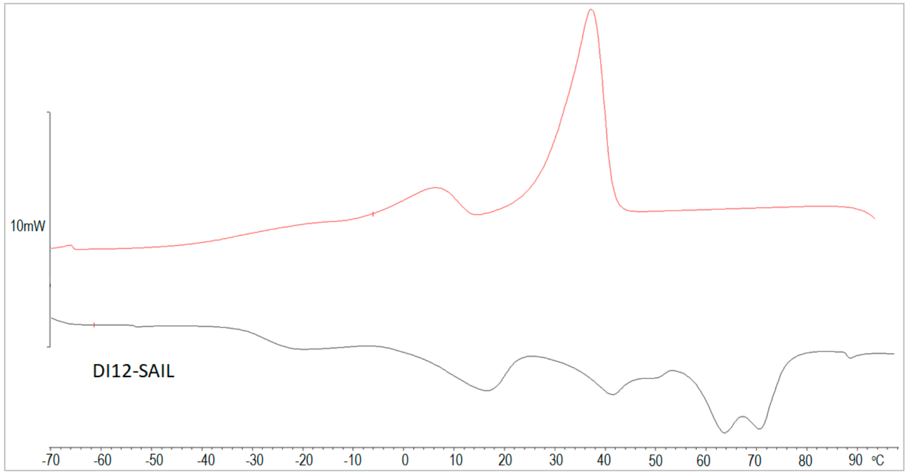

DI$_{12}$-SAIL

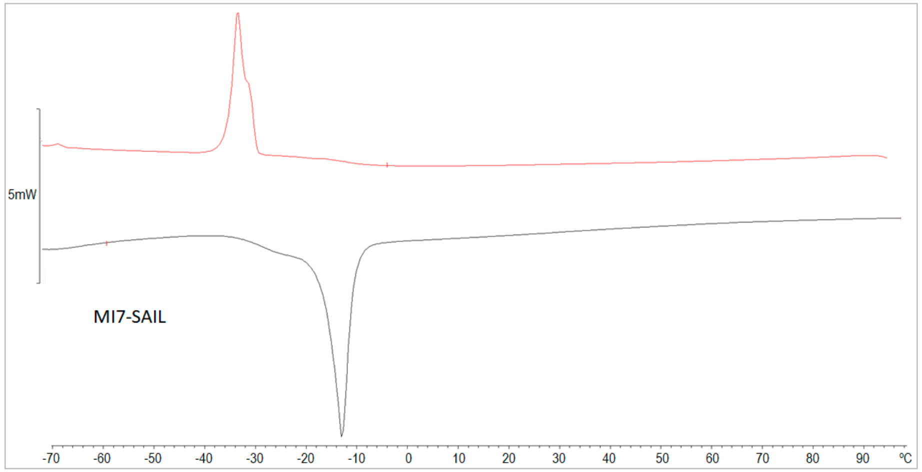

MI$_7$-SAIL

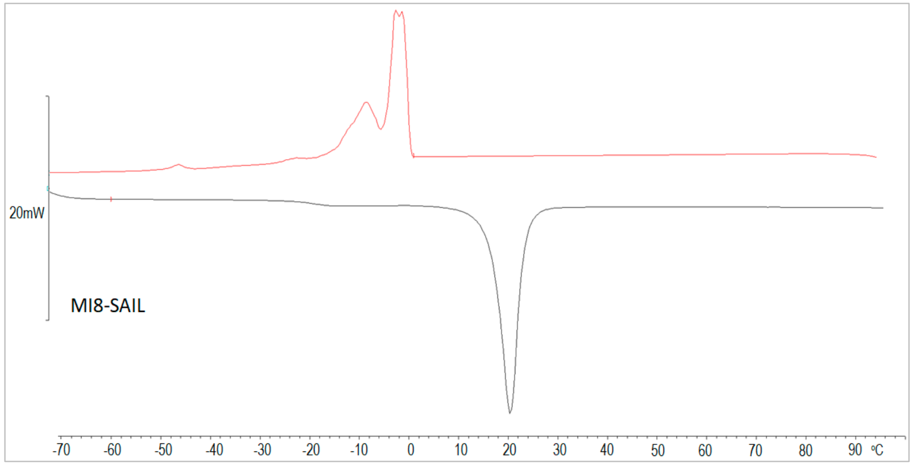

MI$_8$-SAIL

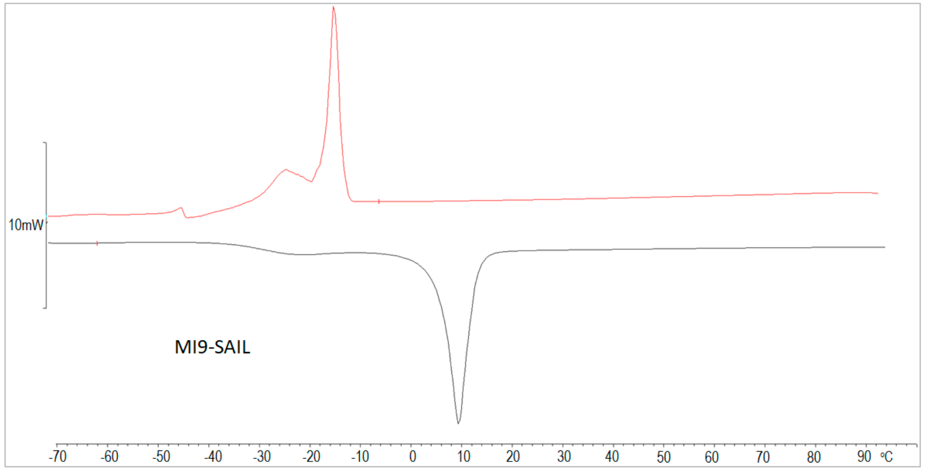

MI$_9$-SAIL

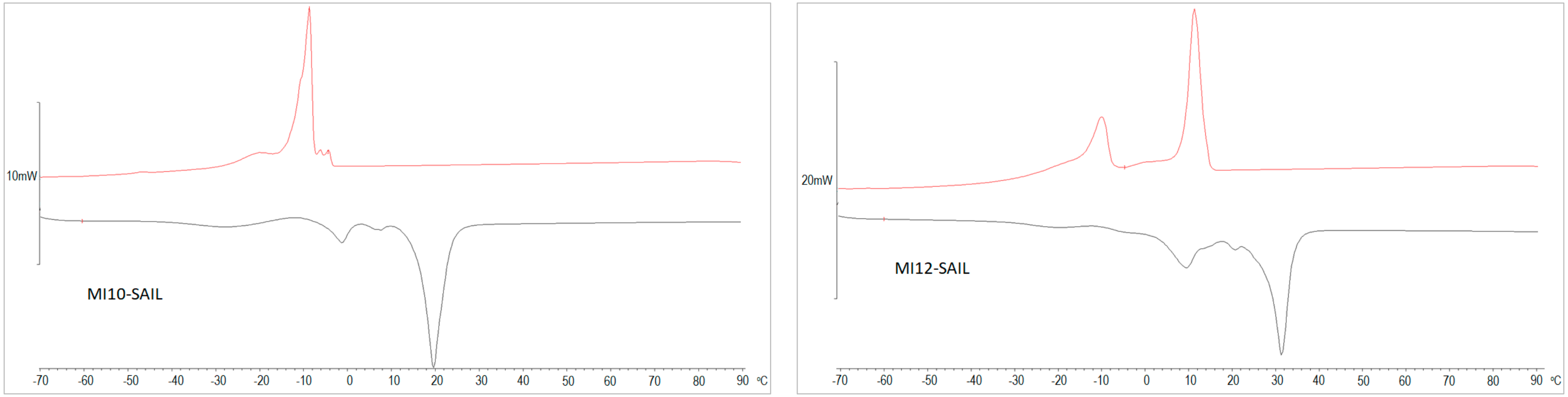

MI$_{10}$-SAIL **MI$_{12}$-SAIL**

Figure S1. DSC thermograms of synthetized SAIL (Black line – heating segment, Red line – cooling segment).

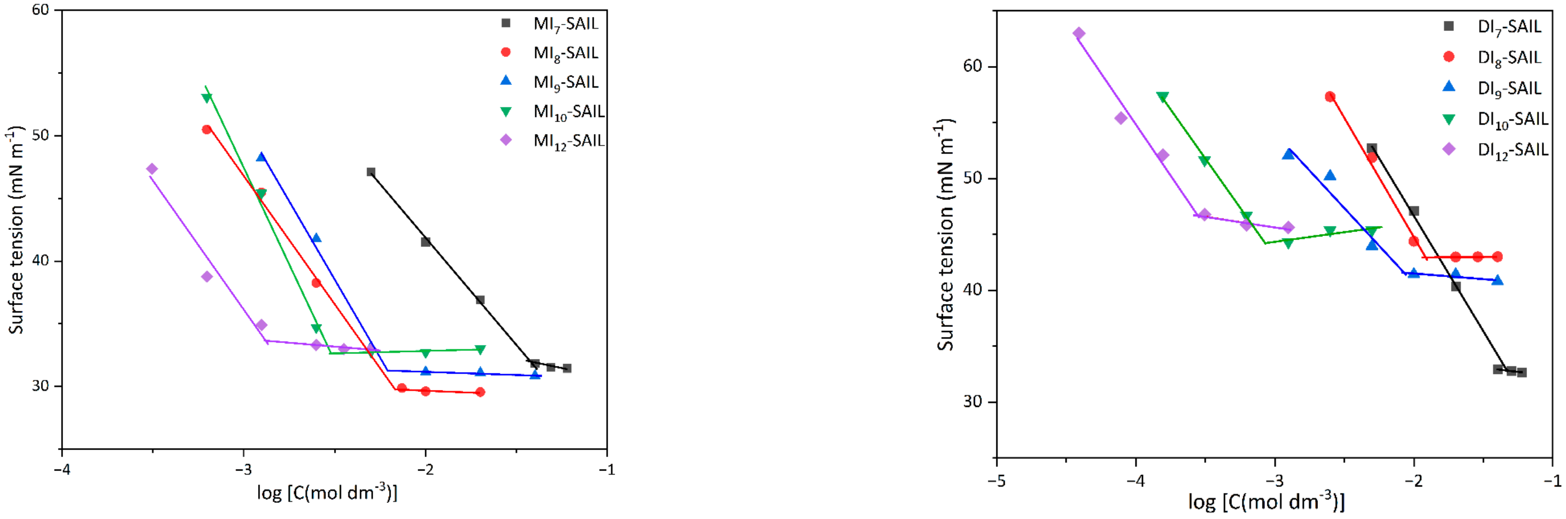

Figure S2. Relationship between surface tension (γ) vs. logarithm of the concentration of synthetized compounds (log C), monocationic SAILs (left side) and dicationic SAILs (right side).

The chemical shifts δ of the studied monocationic and dicationic SAILs are shown below. Hydrogen and carbon atoms are described in Figures S3 and S4 for monocationic and dicationic SAILs respectively. Figures S5-24 show ^{1}H and ^{13}C NMR spectra of the compounds studied.

A monocationic SAIL, where R = C_7H_{15}
^{1}H NMR δ [ppm]: 0.84 (t, $3H_a$), 1.19 (m, $4H_b$), 1.53 (q, $2H_c$), 2.75 (d, $2H_f$), 3.62 (m, $2H_d$), 4.40 (d, $2H_e$), 4.95 (s, $2H_h$), 5.76 (m, $2H_g$), 7.14 (t, $1H_k$), 7.26 (t, $1H_i$), 7.55 (m, $1H_j$), 7.74 (m, $1H_m$), 8.72 (m, $1H_l$), 9.23 (t, $1H_n$)
^{13}C NMR δ [ppm]: 16.06 (CH_3), 24.66 (CH_2), 27.98 (CH_2), 31.32 (CH_2), 31.41 (CH_2), 33.13 (CH_2), 33.81 (CH_2), 51.01 (CH_2), 51.84 (CH_2), 72.41 (CH_2), 81.22 (CH_2), 122.66 (CH), 124.65 (CH), 128.18 (C), 130.82 (CH), 136.77 (CH), 138.31 (CH), 142.94 (CH)

A monocationic SAIL, where R = C_8H_{17}
^{1}H NMR δ [ppm]: 0.93 (t, $3H_a$), 1.26 (m, $6H_b$), 1.51 (q, $2H_c$), 2.76 (d, $2H_f$), 3.60 (m, $2H_d$), 4.42 (d, $2H_e$), 4.89 (s, $2H_h$), 5.77 (m, $2H_g$), 7.06 (t, $1H_k$), 7.22 (t, $1H_i$), 7.53 (m, $1H_j$), 7.74 (m, $1H_m$), 8.74 (m, $1H_l$), 9.29 (t, $1H_n$)
^{13}C NMR δ [ppm]: 16.06 (CH_3), 24.81 (CH_2), 28.26 (CH_2), 31.67 (CH_2), 31.81 (CH_2), 32.00 (CH_2), 32.51 (CH_2), 34.10 (CH_2), 51.06 (CH_2), 51.85 (CH_2), 72.26 (CH_2), 81.28 (CH_2), 122.06 (CH), 124.02 (CH), 128.42 (C), 130.79 (CH), 136.68 (CH), 138.27 (CH), 143.03 (CH)

A monocationic SAIL, where R = C_9H_{19}
^{1}H NMR δ [ppm]: 0.89 (t, $3H_a$), 1.23 (m, $8H_b$), 1.51 (q, $2H_c$), 2.82 (d, $2H_f$), 3.59 (m, $2H_d$), 4.36 (d, $2H_e$), 4.90 (s, $2H_h$), 5.76 (m, $2H_g$), 7.08 (t, $1H_k$), 7.23 (t, $1H_i$), 7.47 (m, $1H_j$), 7.72 (m, $1H_m$), 8.73 (m, $1H_l$), 9.22 (t, $1H_n$)
^{13}C NMR δ [ppm]: 16.13 (CH_3), 24.91 (CH_2), 28.01 (CH_2), 31.58 (CH_2), 31.62 (CH_2), 31.72 (CH_2), 31.81 (CH_2), 32.65 (CH_2), 33.95 (CH_2), 50.99 (CH_2), 51.77 (CH_2), 72.43 (CH_2), 81.21 (CH_2), 122.13 (CH), 124.13 (CH), 128.73 (C), 130.75 (CH), 136.69 (CH), 138.30 (CH), 143.02 (CH)

A monocationic SAIL, where R = $C_{10}H_{21}$
^{1}H NMR δ [ppm]: 0.93 (t, $3H_a$), 1.24 (m, $10H_b$), 1.50 (q, 2Hc), 2.72 (d, $2H_f$), 3.60 (m, $2H_d$), 4.43 (d, $2H_e$), 4.92 (s, $2H_h$), 5.79 (m, $2H_g$), 7.07 (t, $1H_k$), 7.23 (t, $1H_i$), 7.74 (m, $1H_j$), 7.95 (m, $1H_m$), 8.70 (m, $1H_l$), 9.29 (t, $1H_n$)
^{13}C NMR δ [ppm]: 15.80 (CH_3), 24.64 (CH_2), 28.18 (CH_2), 31.55 (CH_2), 31.59 (CH_2), 31.74 (CH_2), 31.86 (CH_2), 31.93 (CH_2), 32.54 (CH_2), 34.07 (CH_2), 50.86 (CH_2), 51.75 (CH_2), 72.08 (CH_2), 81.21 (CH_2), 122.51 (CH), 124.18 (16), 128.37 (C), 130.64 (CH), 136.81 (CH), 137.95 (CH, 143.09 (CH)

A monocationic SAIL, where R = $C_{12}H_{25}$
^{1}H NMR δ [ppm]: 0.76 (t, $3H_a$), 1.06 (m, $14H_b$), 1.29 (q, $2H_c$), 2.58 (d, $2H_f$), 3.39 (m, $2H_d$), 4.22 (d, $2H_e$), 4.71 (s, $2H_h$), 5.58 (m, $2H_g$), 6.84 (t, $1H_k$), 7.01 (t, $1H_i$), 7.15 (m, $1H_j$), 7.54 (m, $1H_m$), 8.42 (m, $1H_l$), 9.10 (t, $1H_n$)
^{13}C NMR δ [ppm]: 15.85 (CH_3), 24.70 (CH_2), 28.13 (CH_2), 31.49 (CH_2), 31.59 (CH_2), 31.65 (CH_2), 31.73 (CH_2), 31.78 (CH_2), 31.87 (CH_2), 31.98 (CH_2), 32.79 (CH_2), 34.03 (CH_2), 50.48 (CH_2), 51.71 (CH_2), 72.09 (CH_2), 81.17 (CH_2), 122.78 (CH), 124.29 (CH), 128.34 (C), 130.53 (CH), 136.89 (CH), 137.89 (CH), 143.05 (CH)

B dicationic SAIL, where R = C_7H_{14}
^{1}H NMR δ [ppm]: 1.26 (m, $6H_a$), 1.54 (q, $4H_b$), 2.73 (t, $4H_c$), 3.60 (t, $4H_e$), 4.29 (t, $4H_f$), 4.88 (s, $4H_d$), 5.60 (s, $4H_g$), 7.26 (t, $2H_h$), 7.31 (t, $4H_i$), 7.41 (t, $4H_j$), 7.66 (t, $2H_k$), 8.65 (m, $2H_l$), 8.99 (t, $2H_m$)
^{13}C NMR δ [ppm]: 27.31 (CH_2), 30.63 (CH_2), 33.96 (CH_2), 51.11 (CH_2), 63.90 (CH_2), 72.60 (CH_2), 80.79 (CH_2), 84.12 (CH_2), 122.29 (CH), 125.11 (CH), 128.53 (C), 130.64 (CH), 130.94 (CH), 137.88 (CH), 142.71 (CH)

B dicationic SAIL, where R = C_8H_{16}
^{1}H NMR δ [ppm]: 1.23 (m, $8H_a$), 1.53 (q, $4H_b$), 2.69 (t, $4H_c$), 3.61 (t, $4H_e$), 4.33 (t, $4H_f$), 4.92 (s, $4H_d$), 5.67 (s, $4H_g$), 7.16 (t, $2H_h$), 7.27 (t, $4H_i$), 7.34 (t, $4H_j$), 7.73 (t, $2H_k$), 8.67 (m, $2H_l$), 9.12 (t, $2H_m$)
^{13}C NMR δ [ppm]: 27.41 (CH_2), 30.72 (CH_2), 30.85 (CH_2), 34.13 (CH_2), 51.69 (CH_2), 63.84 (CH_2), 69.77 (CH_2), 72.61 (CH_2), 80.88 (CH_2), 123.21 (CH), 125.11 (CH), 128.47 (C), 130.30 (CH), 130.82 (CH), 137.98 (CH), 142.79 (CH)

B dicationic SAIL, where R = C_9H_{18}

^{1}H NMR δ [ppm]: 1.19 (m, $10H_a$), 1.52 (q, $4H_b$), 2.68 (t, $4H_c$), 3.62 (t, $4H_e$), 4.38 (t, $4H_f$), 4.91 (s, $4H_d$), 5.66 (s, $4H_g$), 7.13 (t, $2H_h$), 7.26 (t, $4H_i$), 7.34 (t, $4H_j$), 7.70 (t, $2H_k$), 6.86 (m, $2H_l$), 9.07 (t, $2H_m$)

^{13}C NMR δ [ppm]: 27.51 (CH_2), 30.82 (CH_2), 30.93(CH_2), 30.77 (CH_2), 34.21 (CH_2), 51.81 (CH_2), 64.14 (CH_2), 69.76 (CH_2), 72.66 (CH_2), 80.99 (CH_2), 122.95 (CH), 125.21 (CH), 128.59 (C), 130.78 (CH), 131.00 (CH), 138.08 (CH), 142.82 (CH)

B dicationic SAIL, where R = $C_{10}H_{20}$

^{1}H NMR δ [ppm]: 1.20 (m, $12H_a$), 1.54 (q, $4H_b$), 2.77 (t, $4H_c$), 3.61 (t, $4H_e$), 4.32 (t, $4H_f$), 4.89 (s, $4H_d$), 5.67 (s, $4H_g$), 7.11 (t, $2H_h$), 7.23 (t, $4H_i$), 7.33 (t, $4H_j$), 7.53 (t, $2H_k$), 8.66 (m, $2H_l$), 9.10 (t, $2H_m$)

^{13}C NMR δ [ppm]: 27.65 (CH_2), 30.88 (CH_2), 31.09 (CH_2), 31.65 (CH_2), 32.79 (CH_2), 33.91 (CH_2), 50.77 (CH_2), 63.82 (CH2), 69.53 (CH_2), 72.52 (CH_2), 80.96 (CH_2), 122.38 (CH), 123.78 (CH), 128.51 (C), 130.23 (CH), 130.72 (CH), 136.76 (CH), 142.99 (CH)

B dicationic SAIL, where R = $C_{12}H_{24}$

^{1}H NMR δ [ppm]: 1.15 (m, $16H_a$), 1.52 (q, $4H_b$), 2.78 (t, $4H_c$), 3.59 (t, $4H_e$), 4.38 (t, f), 4.91 (s, $4H_d$), 5.71 (s, $4H_g$), 7.15 (t, $2H_h$), 7.21 (t, $4H_i$), 7.35 (t, $4H_j$), 7.71 (t, $2H_k$), 8.66 (m, $2H_l$), 9.17 (t, $2H_m$)

^{13}C NMR δ [ppm]: 27.84 (CH_2), 31.23 (CH_2), 31.45 (CH_2), 31.70 (CH_2), 32.76 (CH_2), 32.96 (CH_2), 34.05 (CH_2), 50.67 (CH_2), 63.75 (CH2), 69.52 (CH_2), 72.18 (CH_2), 81.04 (CH_2), 122.39 (CH), 123.66 (CH), 128.39 (C), 130.58 (CH), 130.93 (CH), 136.70 (CH), 142.94 (CH)

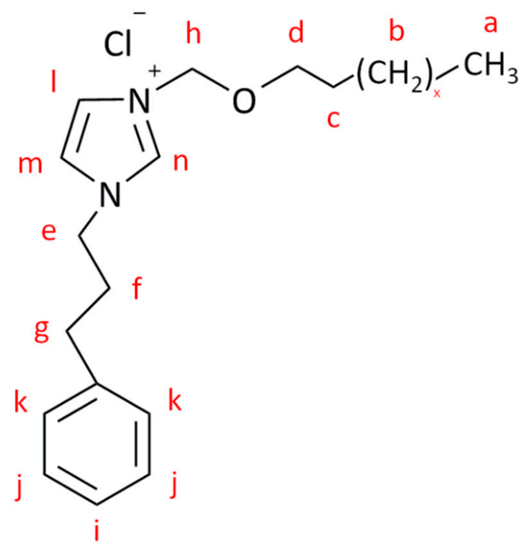

Figure S3. A monocationic SAIL, where x=4 (for R = C_7H_{15}), 5 (C_8H_{17}), 6 (C_9H_{19}), 7 ($C_{10}H_{21}$), and 9 ($C_{12}H_{25}$) with numbered hydrogen atoms.

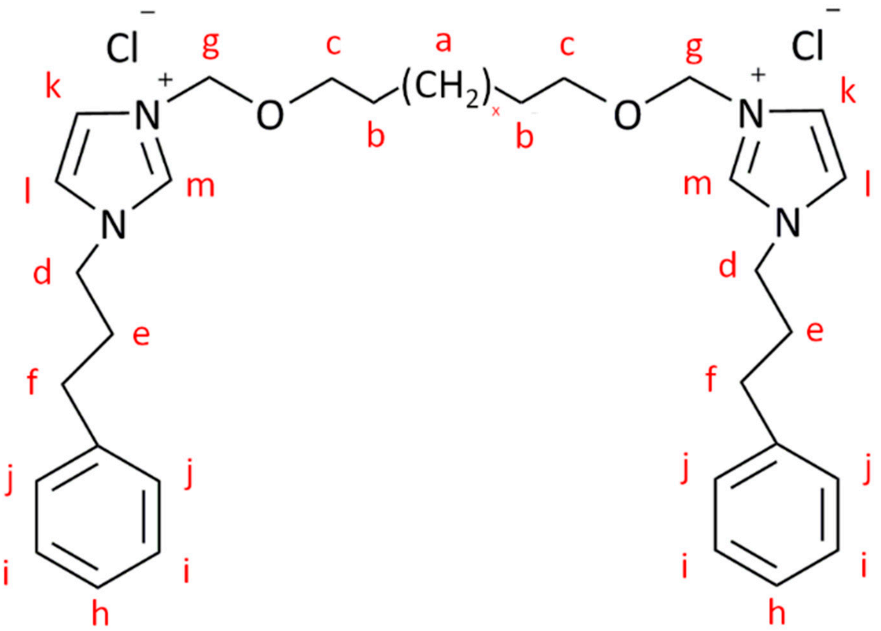

Figure S4. B dicationic SAIL, where x=3 (for R = C_7H_{14}), 4 (C_8H_{16}), 5 (C_9H_{18}), 6 ($C_{10}H_{20}$), and 8 ($C_{12}H_{24}$) with numbered hydrogen atoms.

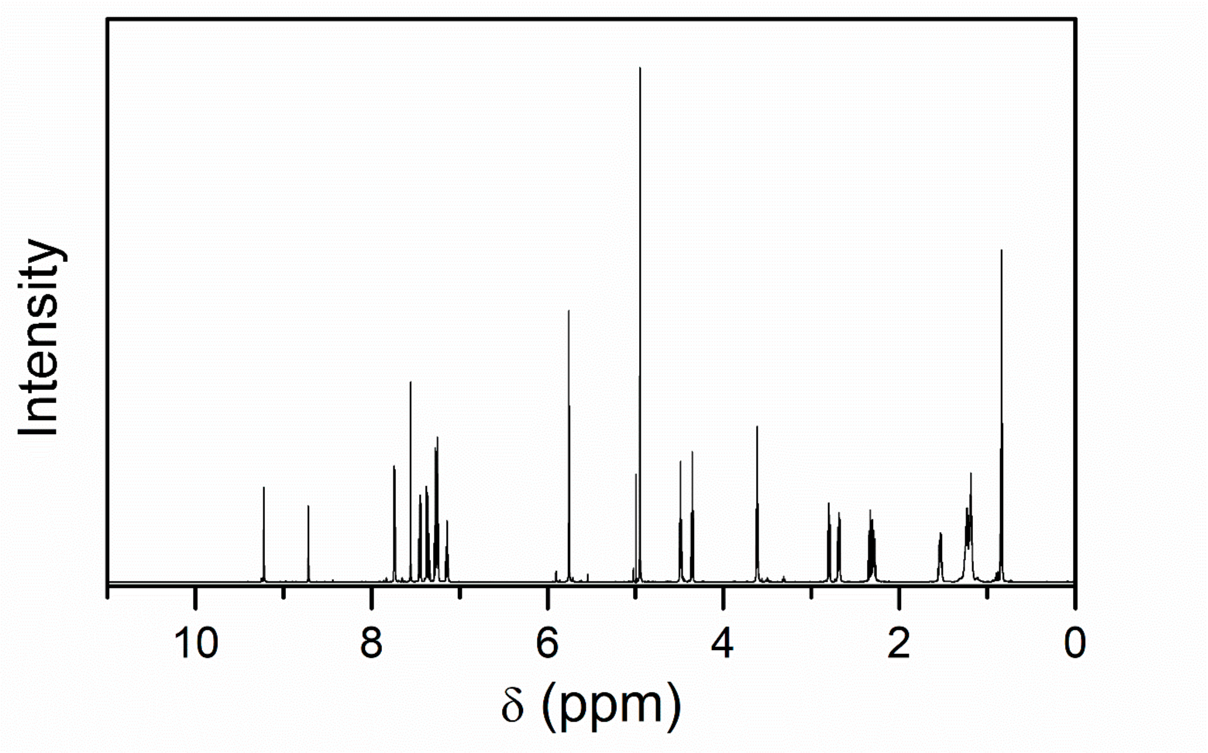

Figure S5. ^{1}H NMR spectrum of the monocationic SAIL, where R = C_7H_{15}.

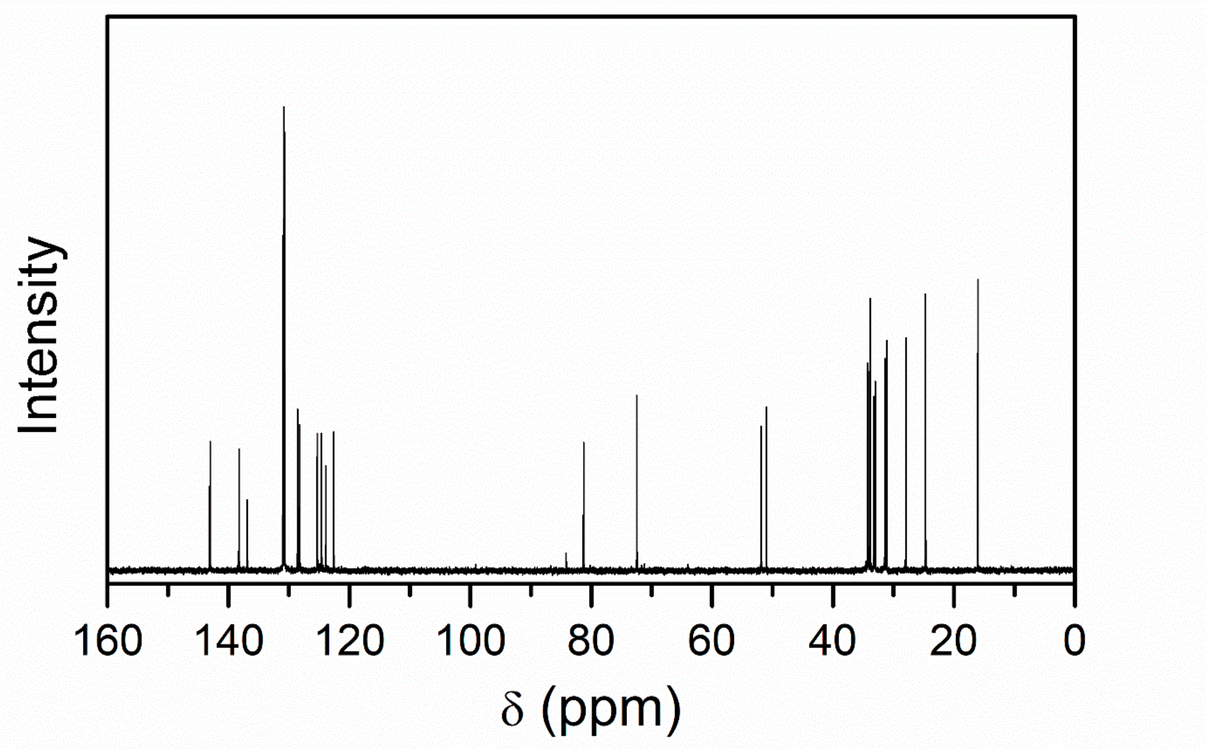

Figure S6. ^{13}C NMR spectrum of the monocationic SAIL, where R = C_7H_{15}.

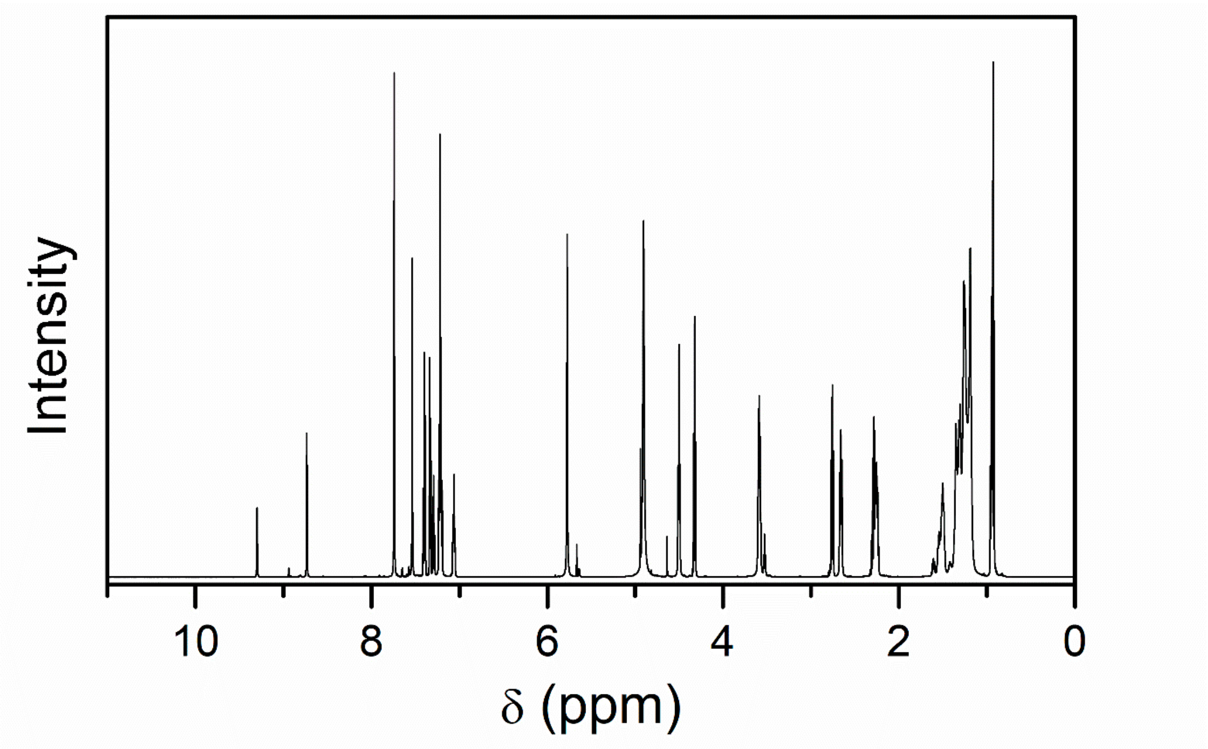

Figure S7. ^{1}H NMR spectrum of the monocationic SAIL, where R = C_8H_{17}.

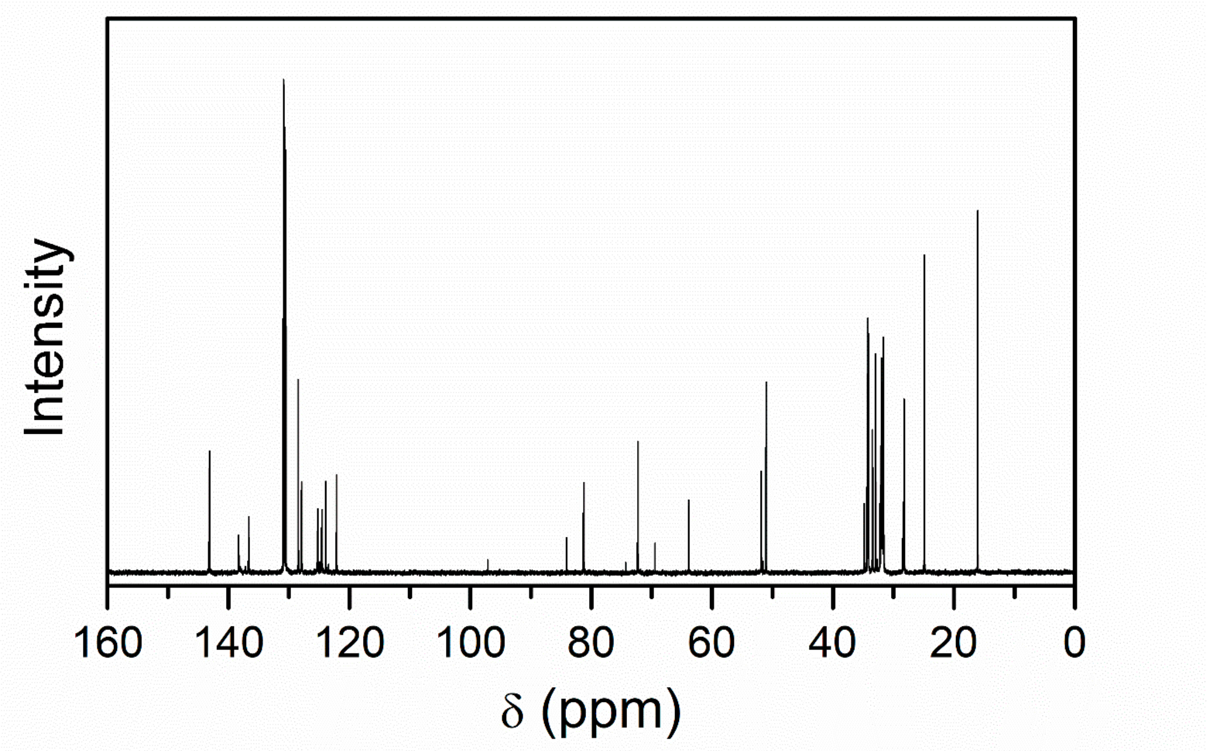

Figure S8. ^{13}C NMR spectrum of the monocationic SAIL, where R = C_8H_{17}.

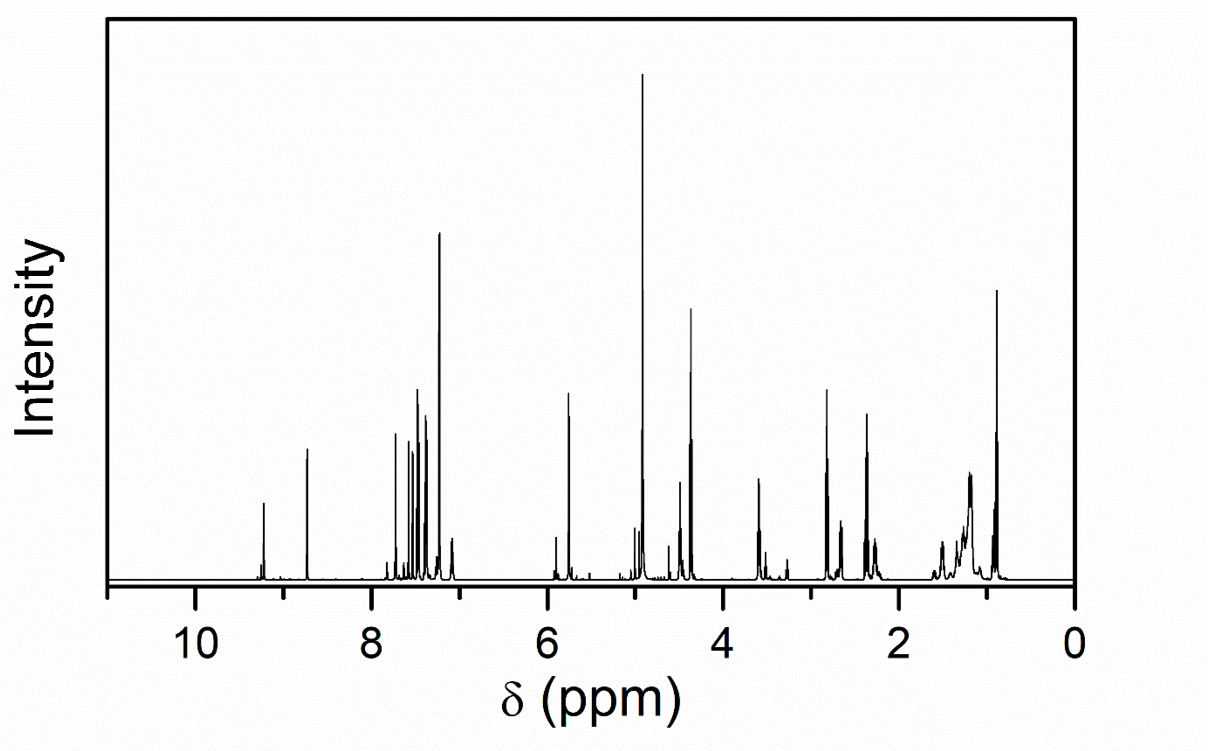

Figure S9. ^{1}H NMR spectrum of the monocationic SAIL, where R = C_9H_{19}.

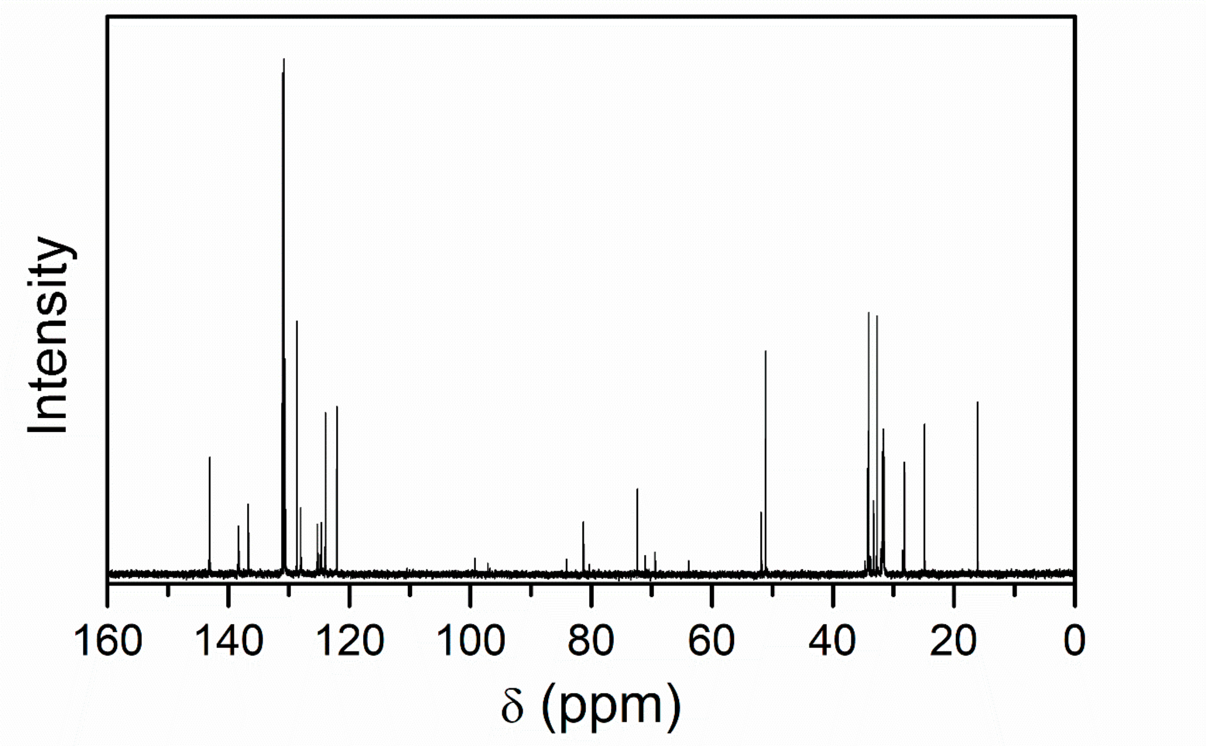

Figure S10. ^{13}C NMR spectrum of the monocationic SAIL, where R = C_9H_{19}.

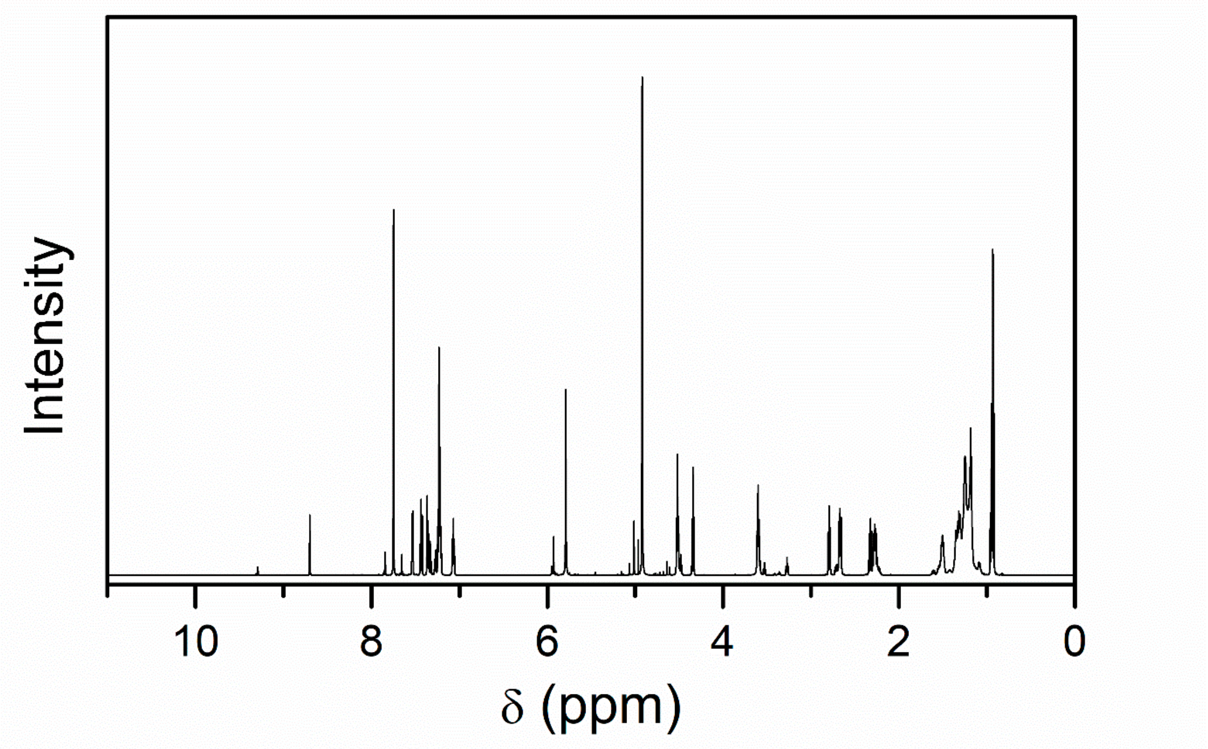

Figure S11. ^{1}H NMR spectrum of the monocationic SAIL, where R = $C_{10}H_{21}$.

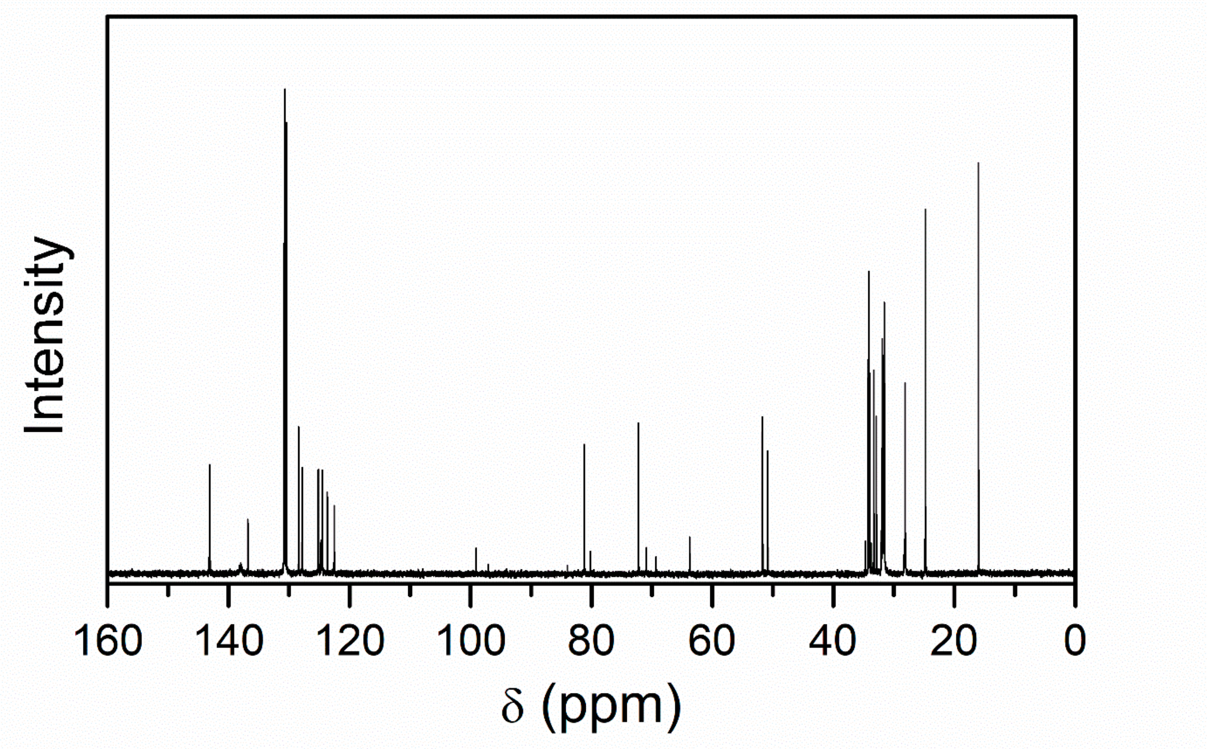

Figure S12. ^{13}C NMR spectrum of the monocationic SAIL, where R = $C_{10}H_{21}$.

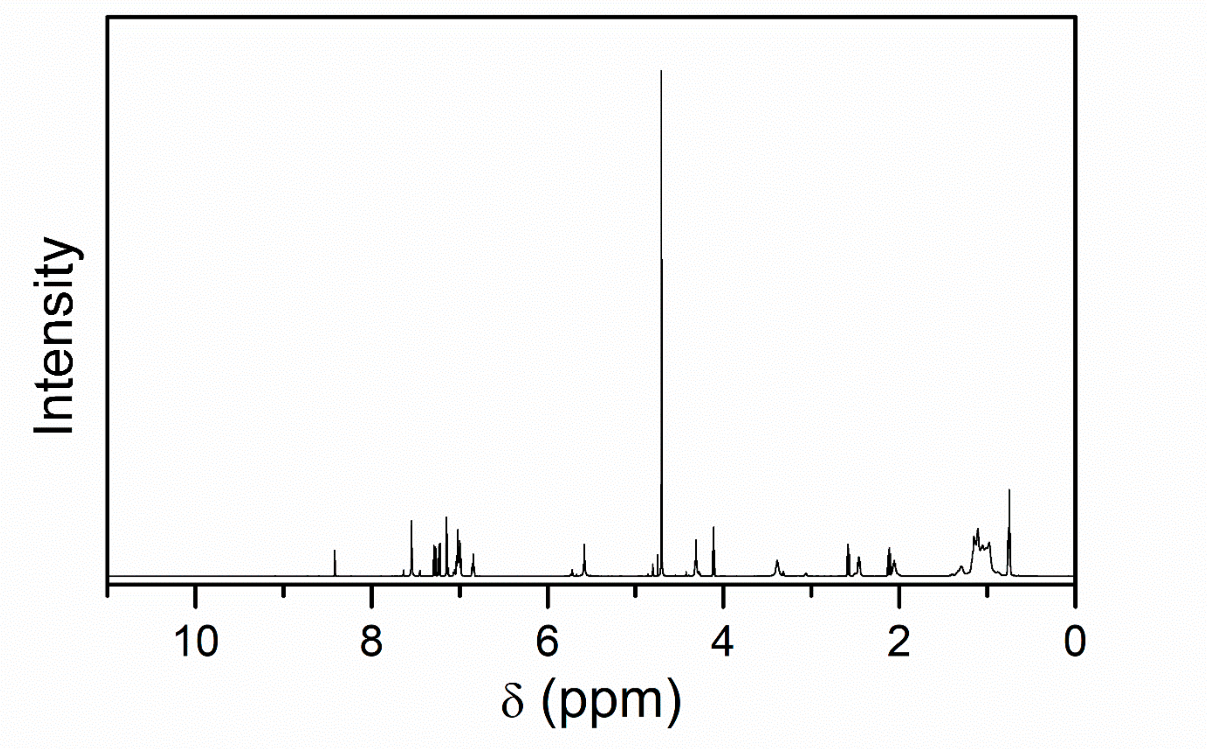

Figure S13. ^{1}H NMR spectrum of the monocationic SAIL, where R = $C_{12}H_{25}$.

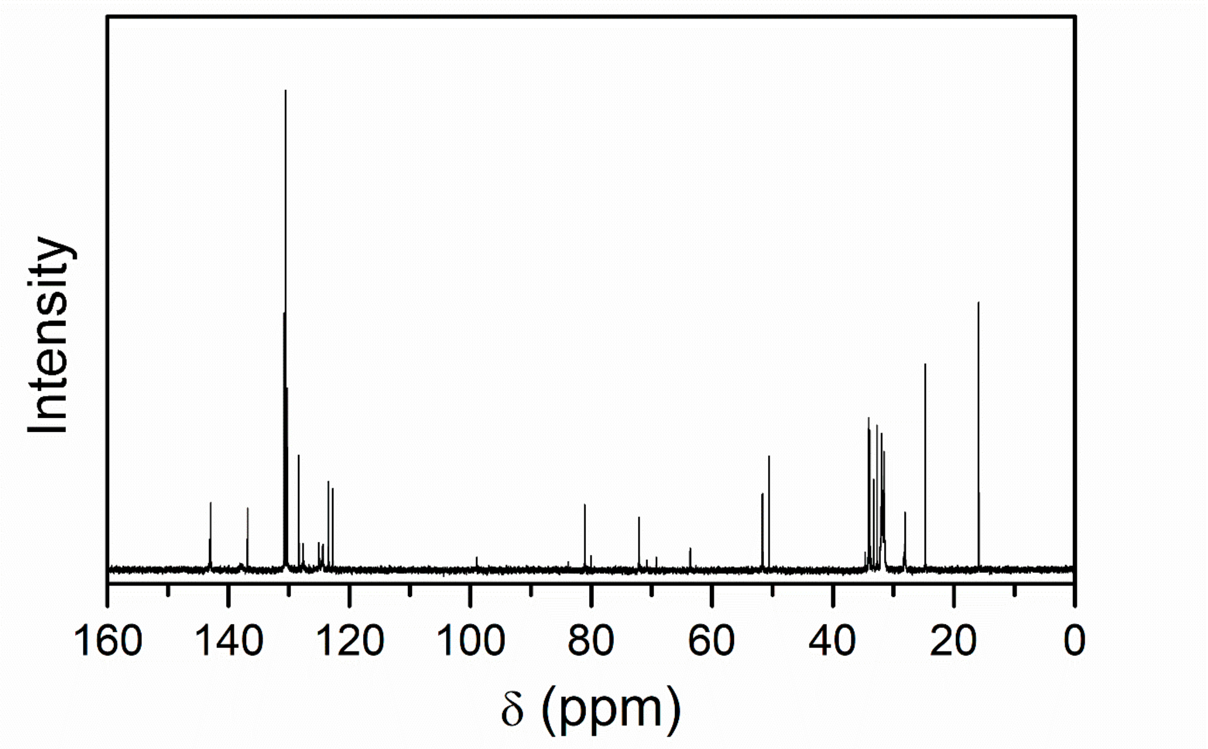

Figure S14. ^{13}C NMR spectrum of the monocationic SAIL, where R = $C_{12}H_{25}$.

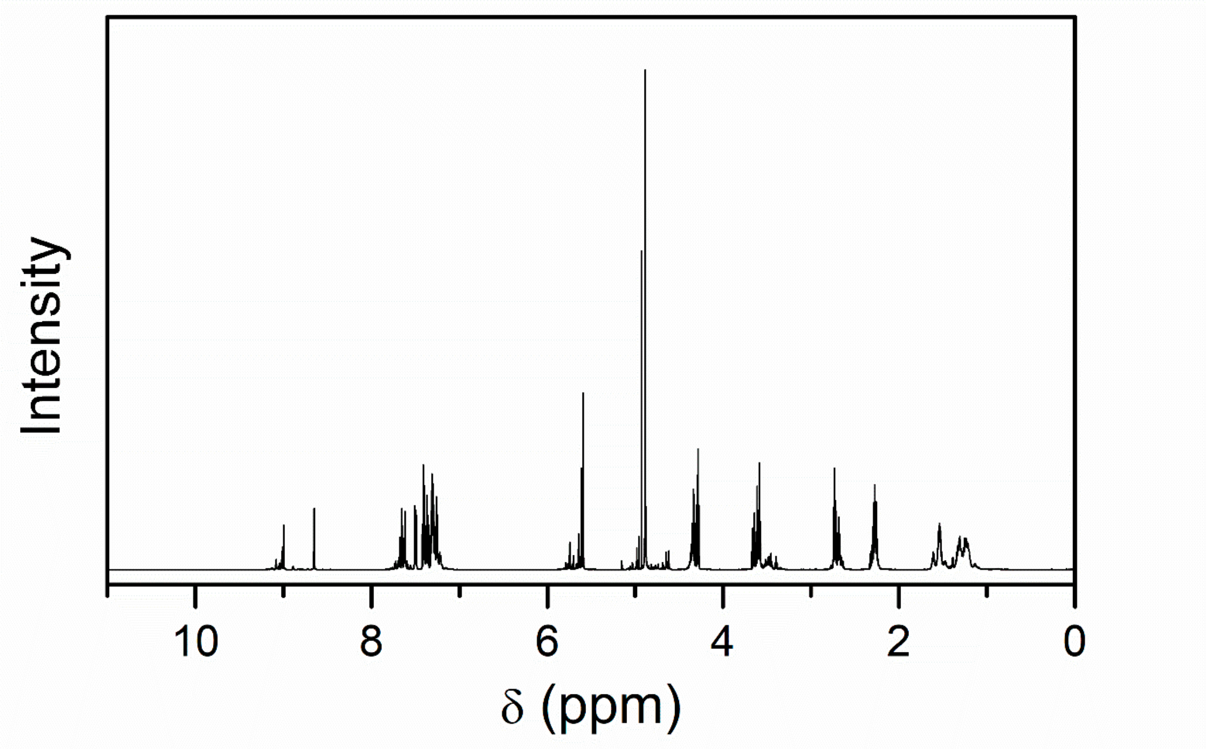

Figure S15. ^{1}H NMR spectrum of the dicationic SAIL, where R = C_7H_{14}.

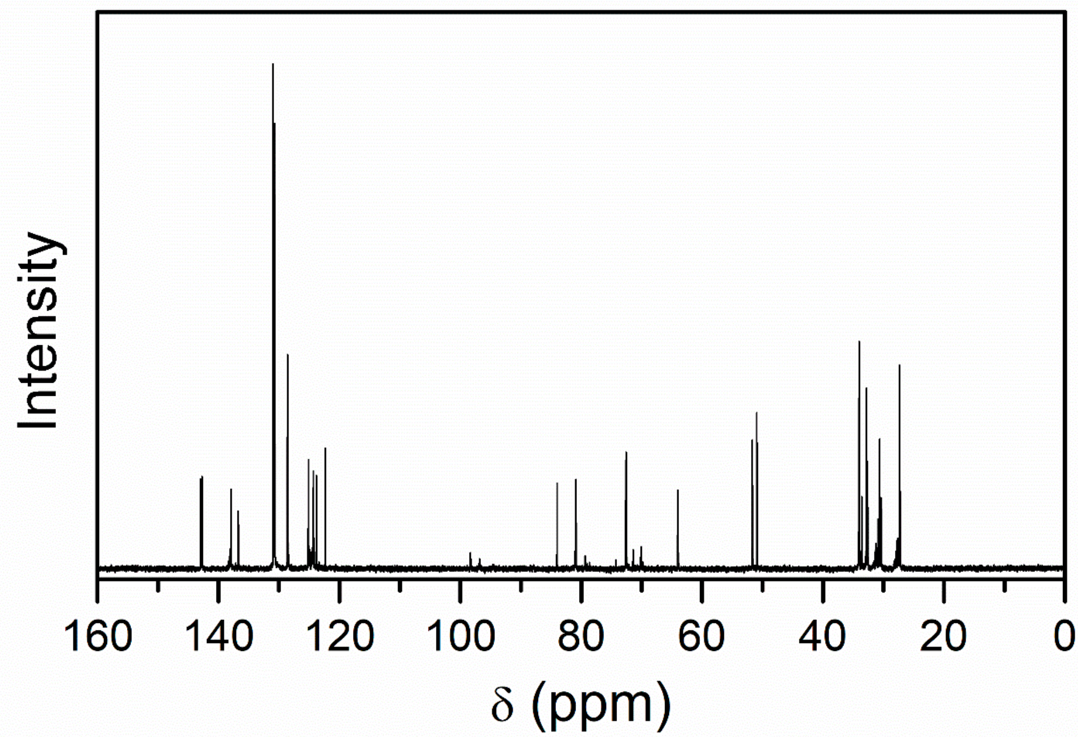

Figure S16. ^{13}C NMR spectrum of the dicationic SAIL, where R = C_7H_{14}.

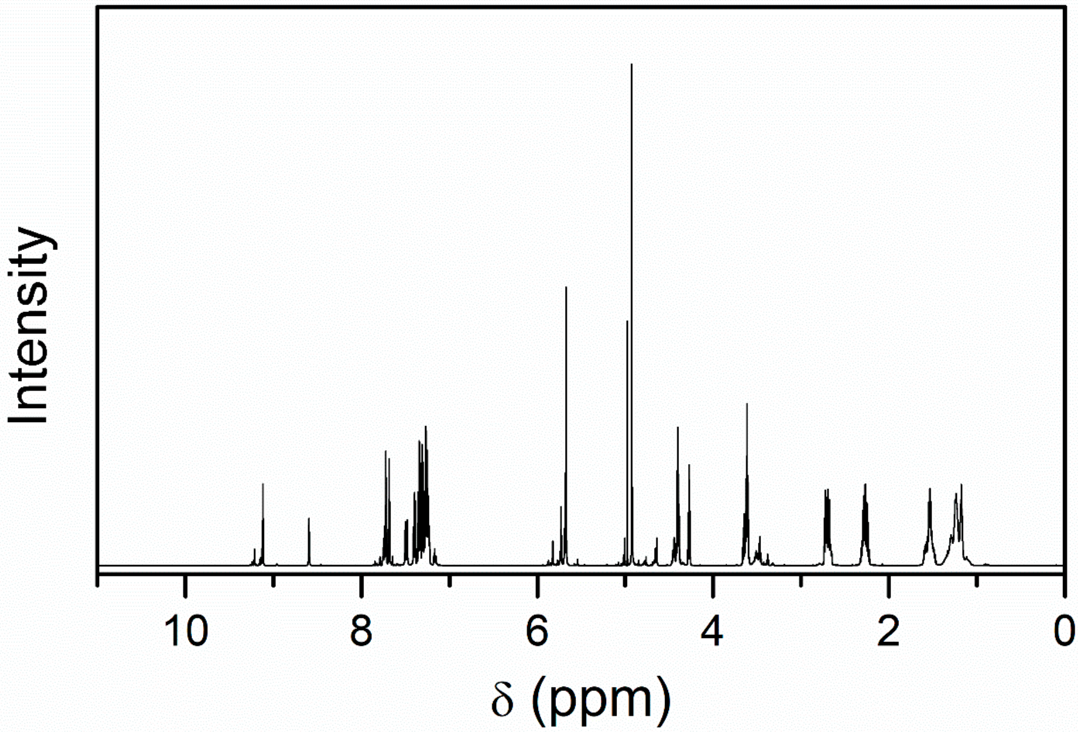

Figure S17. ^{1}H NMR spectrum of the dicationic SAIL, where R = C_8H_{16}.

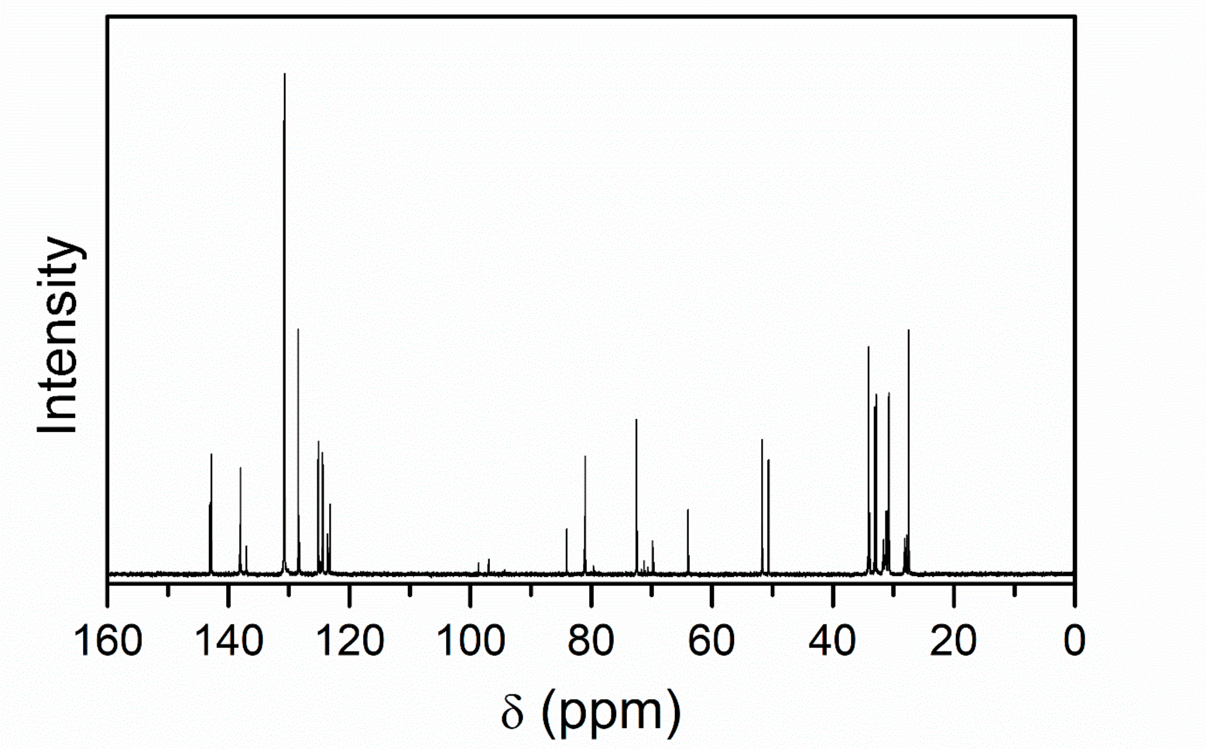

Figure S18. ^{13}C NMR spectrum of the dicationic SAIL, where R = C_8H_{16}.

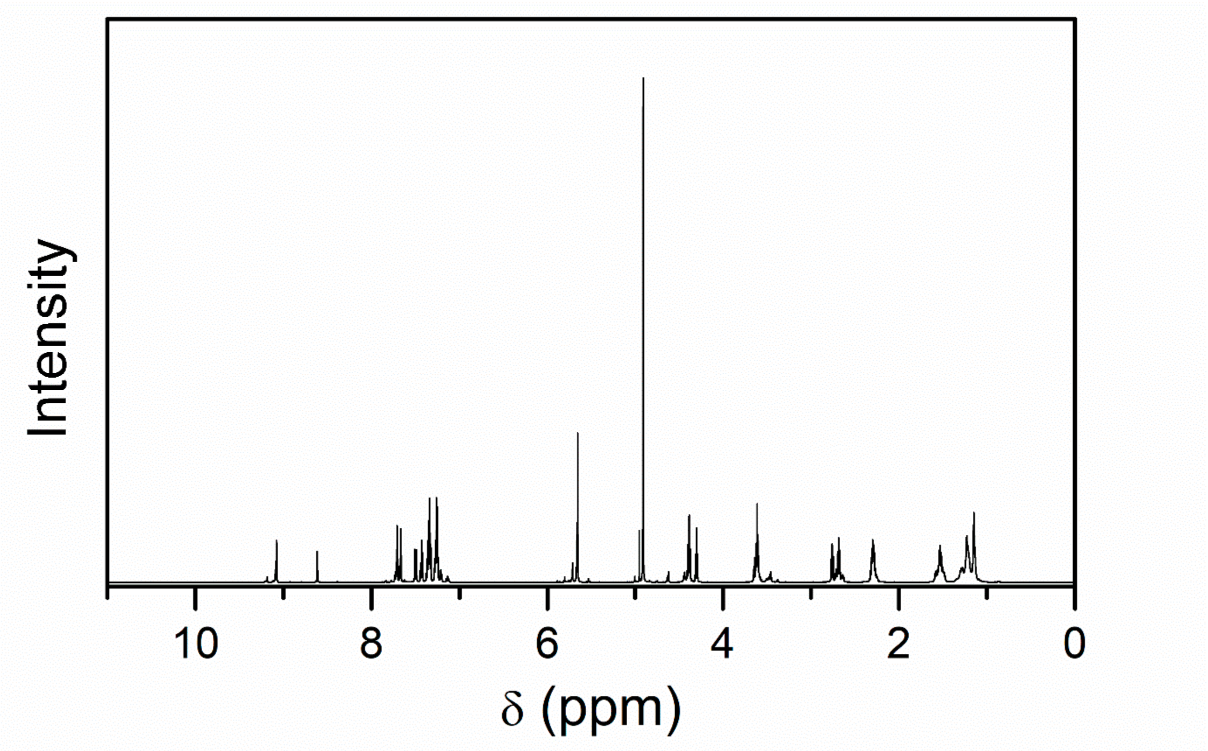

Figure S19. ^1H NMR spectrum of the dicationic SAIL, where R = C_9H_{18}.

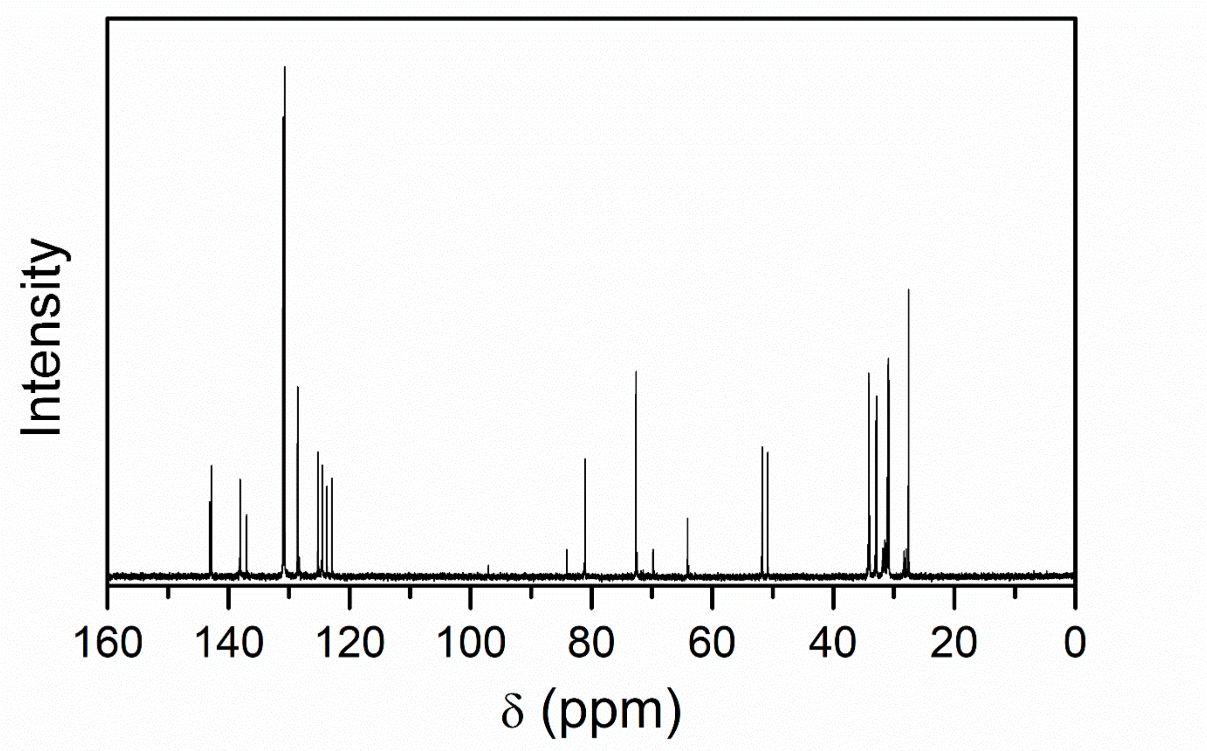

Figure S20. ^{13}C NMR spectrum of the dicationic SAIL, where R = C_9H_{18}.

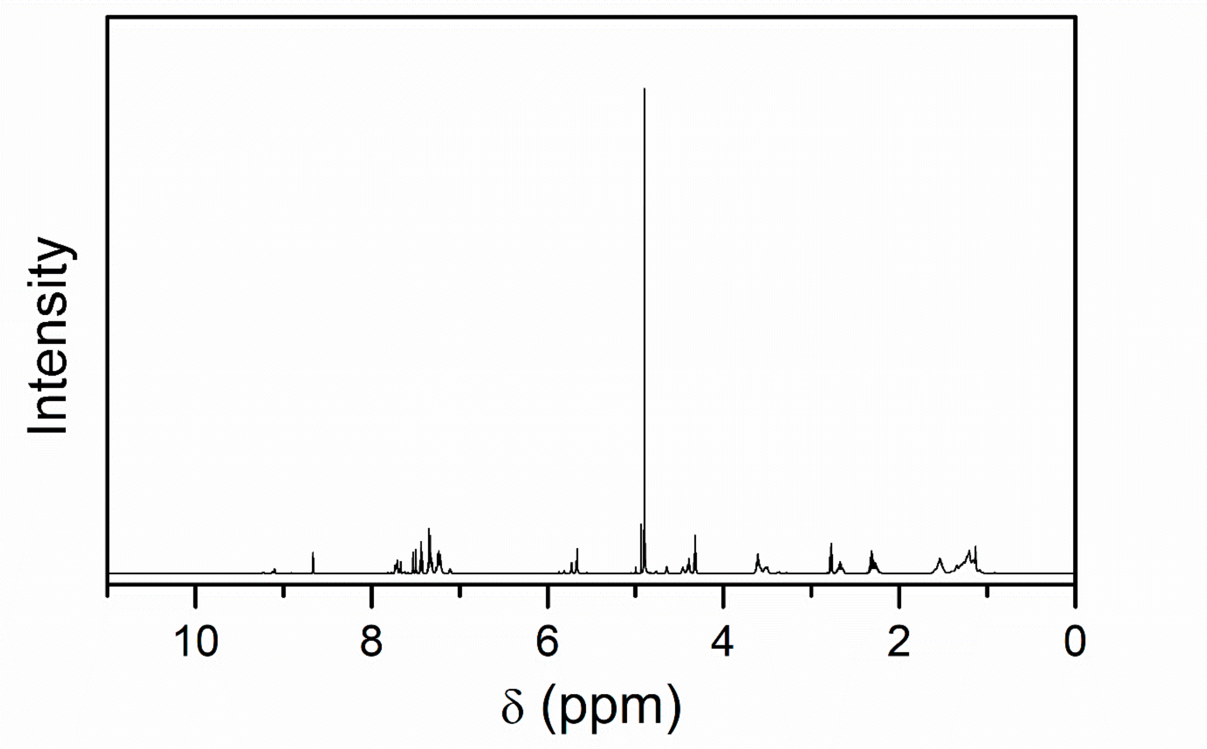

Figure S21. ^{1}H NMR spectrum of the dicationic SAIL, where R = $C_{10}H_{20}$.

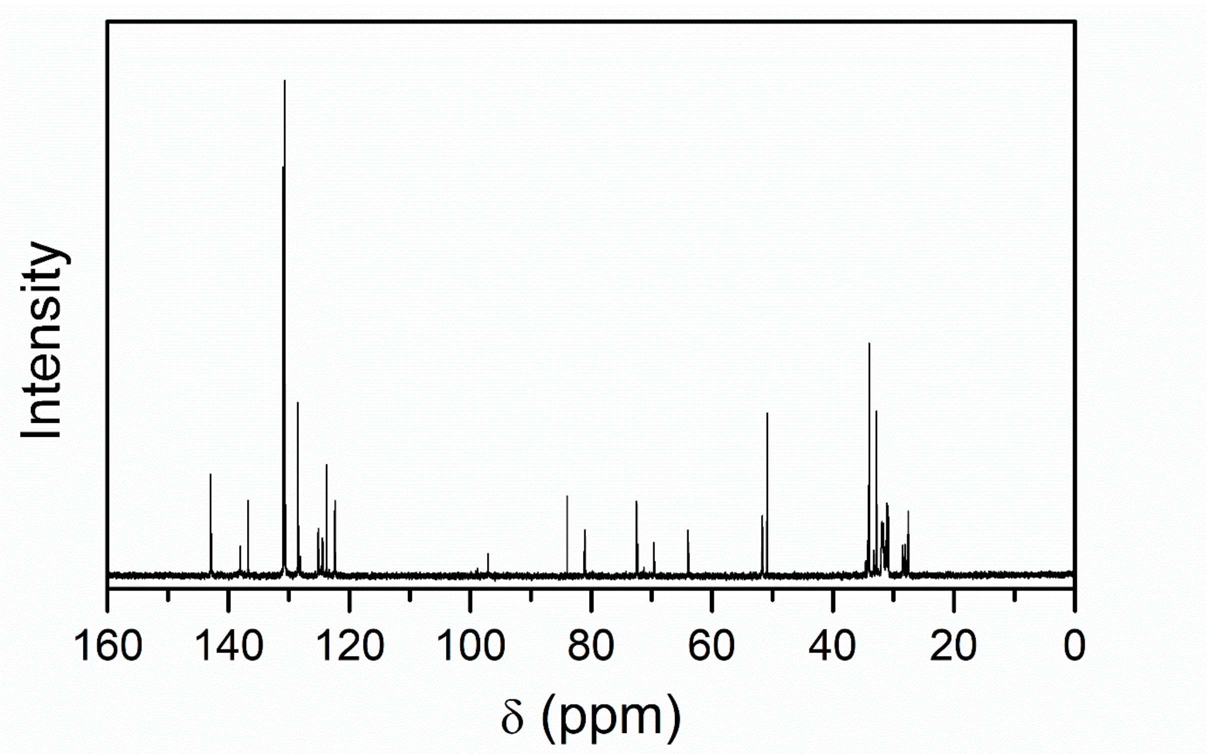

Figure S22. ^{13}C NMR spectrum of the dicationic SAIL, where R = $C_{10}H_{20}$.

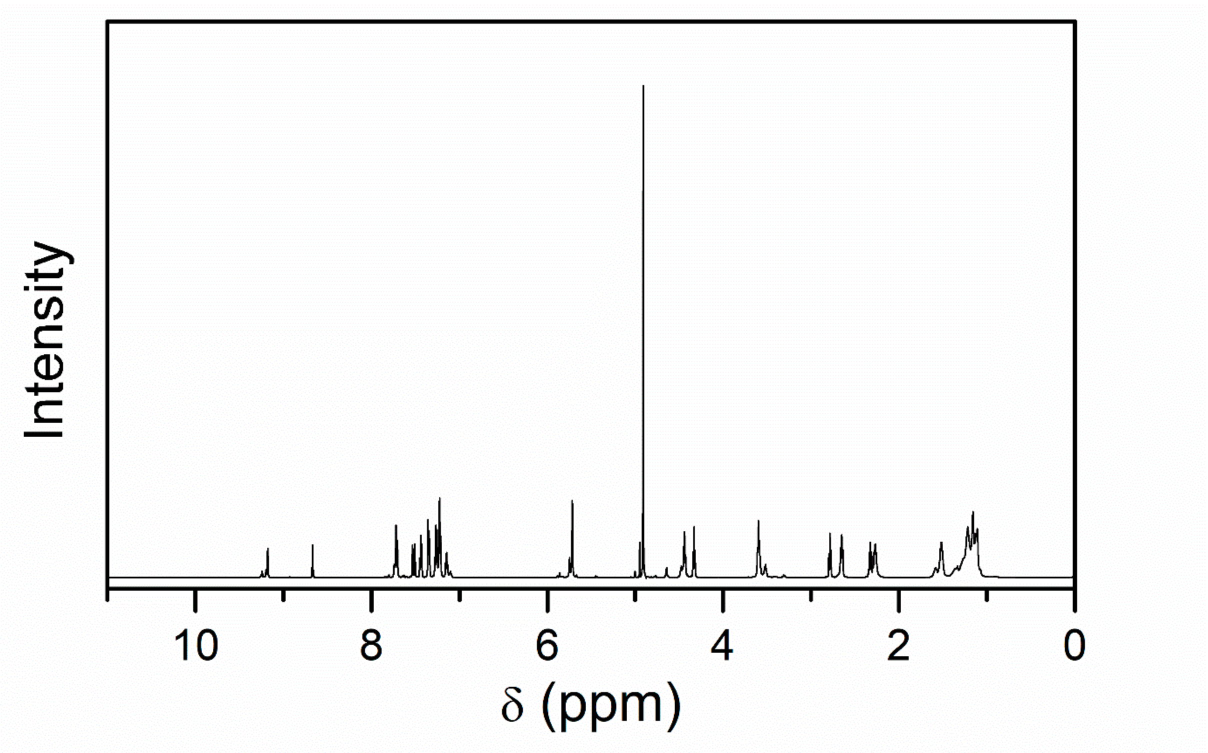

Figure S23. ^{1}H NMR spectrum of the dicationic SAIL, where R = $C_{12}H_{24}$.

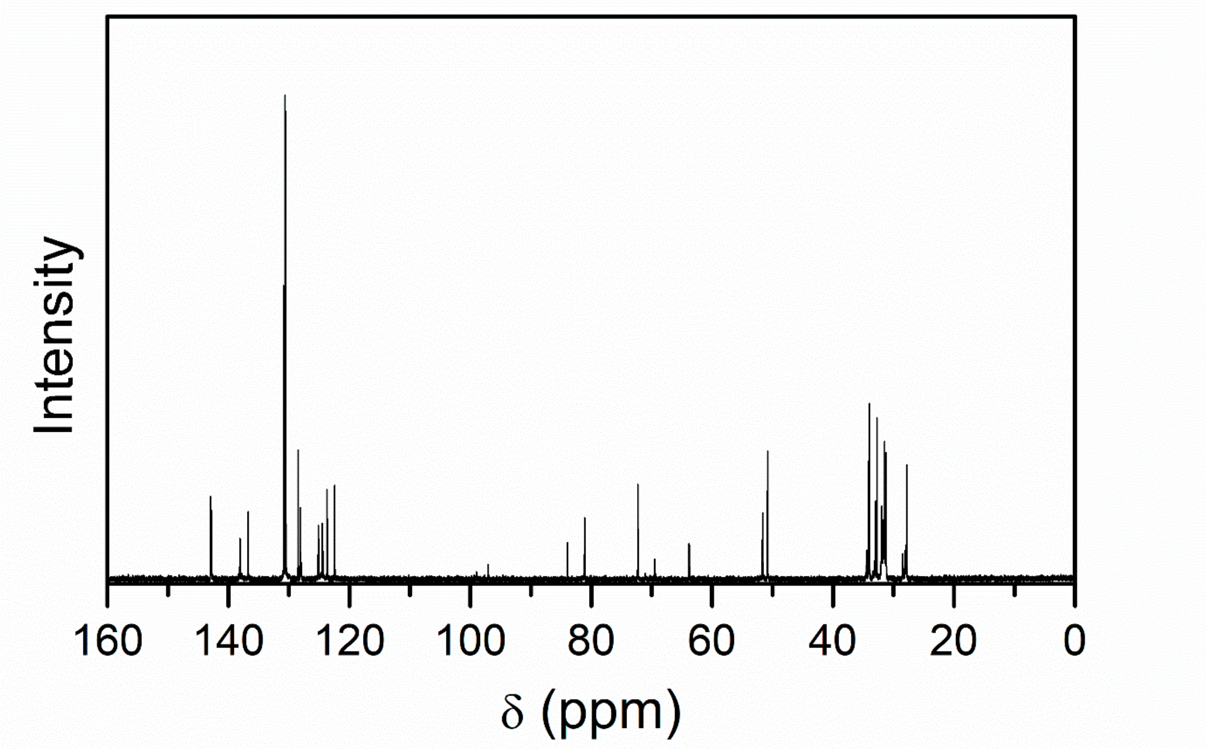

Figure S24. ^{13}C NMR spectrum of the dicationic SAIL, where R = $C_{12}H_{24}$.
